# The neural basis of naturalistic semantic and social cognition

**DOI:** 10.1038/s41598-024-56897-3

**Published:** 2024-03-21

**Authors:** Melissa Thye, Paul Hoffman, Daniel Mirman

**Affiliations:** Department of Psychology, University of Edinburgh, Edinburgh, EH8 9JZ UK

**Keywords:** Language, Social behaviour

## Abstract

**Abstract:**

Decoding social environments and engaging meaningfully with other people are critical aspects of human cognition. Multiple cognitive systems, including social and semantic cognition, work alongside each other to support these processes. This study investigated shared processing between social and semantic systems using neuroimaging data collected during movie-viewing, which captures the multimodal environment in which social knowledge is exchanged. Semantic and social content from movie events (event-level) and movie transcripts (word-level) were used in parametric modulation analyses to test (1) the degree to which semantic and social information is processed within each respective network and (2) engagement of the same cross-network regions or the same domain-general hub located within the semantic network during semantic and social processing. Semantic word and event-level content engaged the same fronto-temporo-parietal network and a portion of the semantic hub in the anterior temporal lobe (ATL). Social word and event-level content engaged the supplementary motor area and right angular gyrus within the social network, but only social words engaged the domain-general semantic hub in left ATL. There was evidence of shared processing between the social and semantic systems in the dorsolateral portion of right ATL which was engaged by word and event-level semantic and social content. Overlap between the semantic and social word and event results was highly variable within and across participants, with the most consistent loci of overlap occurring in left inferior frontal, bilateral precentral and supramarginal gyri for social and semantic words and in bilateral superior temporal gyrus extending from ATL posteriorly into supramarginal gyri for social and semantic events. These results indicate a complex pattern of shared and distinct regions for social and semantic cognition during naturalistic processing.

**Protocol registration:**

The stage 1 protocol for this Registered Report was accepted in principle on October 11, 2022. The protocol, as accepted by the journal, can be found at: 10.17605/OSF.IO/ACWQY.

## Introduction

Social knowledge is a fundamental aspect of human cognition: it informs our moment-by-moment understanding of our social world, and it directly motivates human behaviour^1^. Through our understanding of social concepts and behaviours we are able to effectively and accurately communicate complex, abstract ideas and participate in meaningful interpersonal interactions. Many of these processes are, at least partially, supported by the social cognition system, which is broadly engaged in integrating and updating information about the actions, beliefs, motives, and mental states of ourselves and the people in our environment. Much of the research on social knowledge has focused on characterizing how individuals engage the social cognition system to process information about their own and others’ actions and perspectives^2^. Decoding and reciprocating interactional dynamics leverages a whole host of other cognitive systems, ranging from perceptual and attentional systems to higher-order language and executive systems^3^. Shifting from a strict domain specificity approach and adopting models from other domains of cognition may result in greater insights in social neuroscience^4,5^. One key contributor is semantic cognition, which allows us to know and communicate about both the linguistic and non-linguistic physical and emotional properties of the objects, people, and events we experience, and gives meaning to the language we use^6–9^. The breadth and complexity of social knowledge requires mutual or interacting support from both social and semantic cognition, and the present study examines the degree to which social cognitive processing leverages the neural architecture of the semantic system.

A rich history of research on pragmatics shows that social cognition plays an important role in communication, where context and non-linguistic features convey critical information that is not present in the lexical units or syntactic structures themselves. This pragmatic content allows comprehension of the intended meaning beyond the surface linguistic content^10,11^ and requires the social cognitive process of mentalizing about the perspectives of the other agents in the environment^12^. Retrieval of the relevant social knowledge—from the names and behaviours of the people we encounter to the concepts used to label those behaviours—may rely on semantic memory, which is an acquired conceptual store of linguistic and non-linguistic information about the multimodal world around us, informed by interactions with new objects, events, experiences, and people^7,8,13^. Although pragmatics is predominately concerned with how communicative intent is inferred in the presence or absence of linguistic input which is separate from the goals of the present study, the research in this domain emphasizes the importance of the social cognition system in communication, a role which is also facilitated by semantic memory.

One clear point of intersection between semantic and social cognition is the representation and processing of social concepts. What makes these concepts ‘social’ is their use in ascribing meaning to human behaviour, intentions, desires, feelings, and interactions^14^. This type of social knowledge is often (although not universally^15^) described as intangible or abstract, not grounded in sensory or perceptual representations^16^. Current neurocomputational theories posit that abstract semantic representations arise through statistical regularities in the contexts in which they occur, especially natural language contexts. Concepts such as *jealousy* and *ambition* co-occur with concrete concepts in specific contexts, and knowledge about our own and others’ emotions, intentions, and beliefs is encoded along with the environment in which they occurred, thus giving rise to abstract social concepts^17–19^. Social concepts, like other types of semantic knowledge^14,20^, are acquired through interactions in social environments in which individuals display or communicate about the behaviours associated with these concepts. As a result, these concepts are predominately not understood through sensory systems, and are instead directly informed by and grounded in emotion^21^, introspection^22^, and social experiences^19^. The roles of semantic and social cognition in acquiring social knowledge are thus inseparable.

**Figure 1:**
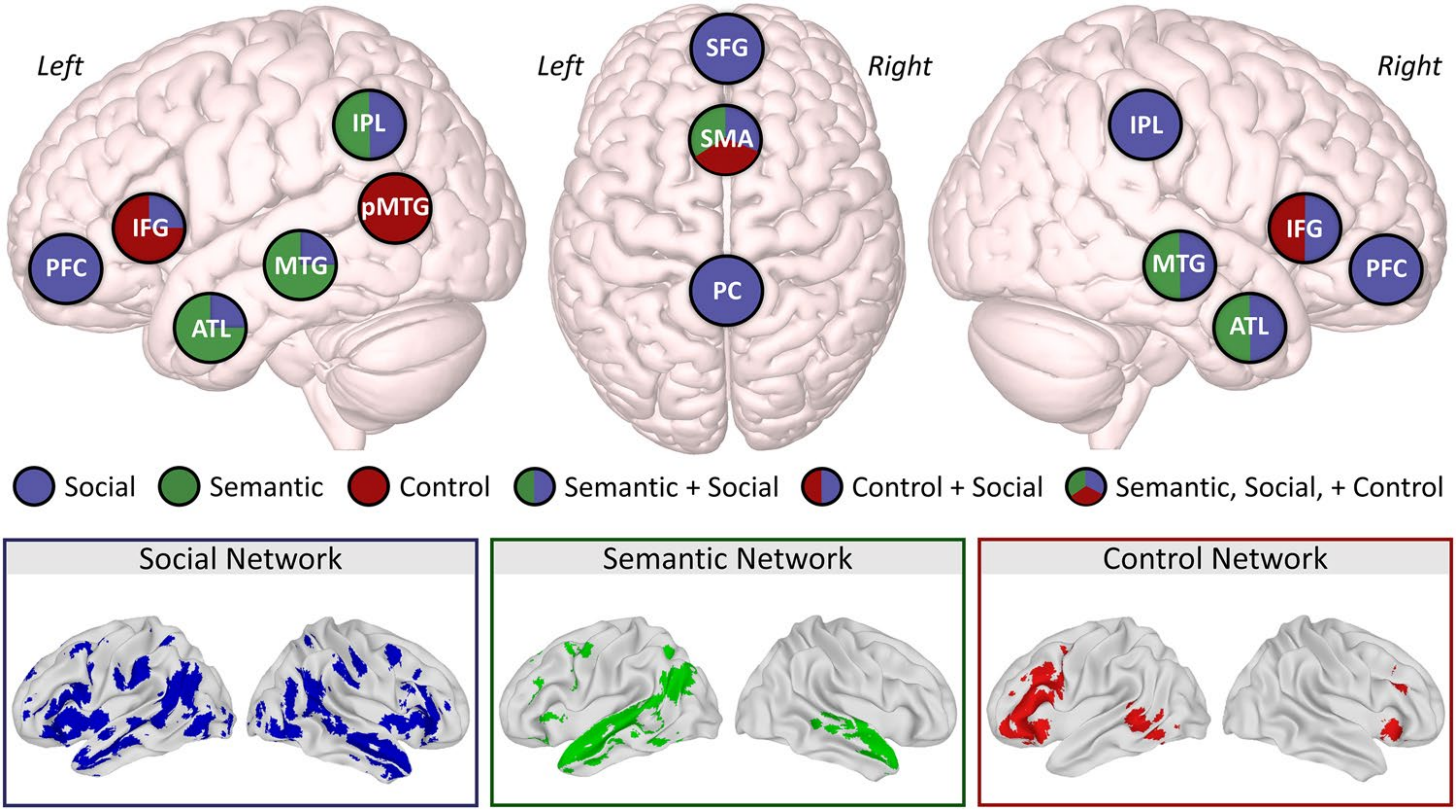
Social, semantic, and semantic control brain networks. A schematic showing the critical regions within the social network (blue), semantic network (green), and semantic control network (red) is shown in the top panel. The overlap between the regions within each network is indicated by circles with mixed colours, and the relative extent of overlap is shown by the amount of colour associated with a given network in each circle (either approximately equally shared—indicated with ½–½ shading—or predominately reported for one system with a smaller subset of the region reported for the other system—indicated with ¾–¼ shading). The statistical maps derived from coordinate-based activation likelihood estimation (ALE) analyses of social cognition^31^ and semantic cognition and semantic control^39^ are shown in the bottom panel. ATL, anterior temporal lobe; IFG, inferior frontal gyrus; IPL, inferior parietal lobule; MTG, middle temporal gyrus; PC, precuneus; PFC, prefrontal cortex; pMTG, posterior middle temporal gyrus; SFG, superior frontal gyrus; SMA, supplementary motor area.

In addition to shared conceptual knowledge, the semantic and social systems are supported by an overlapping network of brain regions (Fig. 1)^3,7,23^. This overlap predominately occurs in the anterior temporal lobes (ATL) and the left inferior parietal lobule, regions which are consistently reported in semantic processing^24,25^, including representing and retrieving social knowledge^14,26,27^, and in mentalizing tasks^28,29^. Engagement of the same regions for semantic and social processing has motivated a theoretical account, the *graded semantic hub hypothesis*, which argues that social cognition requires semantic memory and the neural architecture of the semantic cognition and semantic control systems^7,20,30^. The same ventrolateral portion of left ATL is engaged by theory of mind processing and non-verbal semantic processing^30^, and a recent meta-analysis found that both cognitive systems rely on a shared cognitive control network^31^, which provides empirical support for this account. In addition, the ATL may be ideally positioned to serve as a hub for processing both semantic and social information given the structural connections of the uncinate fasciculus projecting from ATL to emotion processing areas in amygdala and orbitofrontal cortex^26^. Notwithstanding this evidence of overlap, there is also extensive evidence that the networks that support language processing and theory of mind processing are separable^32,33^. Compelling evidence of this dissociation comes from lesion studies in which individuals with extensive left hemisphere damage or aphasia have preserved theory of mind processing or ability to comprehend communicative intent^34,35^. This dissociation is not observed in patients with semantic dementia, which is characterized by bilateral ATL damage, who display impairments in both semantic and social processing^36^. This suggests that the location of the damage (i.e., whether the damage occurs in a shared ATL processing hub) may determine whether only one or both systems are affected. There is thus ample evidence that language and social processing can be dissociated, but a focus on separability ignores potential insights about interactive processing^37,38^ and cannot answer whether the regions engaged by both systems are responding to both types of content (i.e., semantic and social processing) or serving as domain-general hubs that support both processes. In this view, specialization, and therefore dissociation, does occur for semantic and social processing which separately recruit more specialized regions outside of these hubs.

Overlapping neural networks may simply indicate that the semantic and social systems work alongside each other, with areas of overlap performing separate functions within each system (no shared processing) or there may be non-overlapping specialized sub-regions for processing semantic or social information (graded functioning). Alternatively, domain-general areas may perform the same function within each system (shared processing) or a known semantic or social hub may integrate information to facilitate processing both semantic and social information (shared hub). One reason to expect shared processing or a shared hub is that semantic and social cognition have been proposed to consist of analogous representation and control processes. Control processes for both systems are supported by a shared control network^31^, and the hub-and-spoke sensorimotor architecture of the semantic representation system lends itself well to multimodal social perceptual stimuli^7^. Recent research provides critical evidence in support of this claim^30^, but the social and semantic tasks used were highly simplified and thus impoverished approximations of real-world cognition. Also, the inferences were drawn from the group-level statistical map rather than overlap at the individual participant level. Stronger evidence of shared processing or a shared hub would come from using naturalistic paradigms and investigating the neural overlap of these systems within individuals because idiosyncratic variations in neuroanatomy and functioning are ignored when aggregating results at the group-level^40^. The latter is especially relevant when studying regions that may have graded functioning. Previous research attempting to isolate theory of mind (i.e., false belief) and linguistic (non-social stories) processing within the superior temporal sulcus (STS) found overlapping voxels in both posterior and anterior portions of STS that responded to both types of content within individuals^41^. These results suggest that the neural overlap reported in group studies is capturing meaningful overlap that exists within subjects, but this study did not test the broader semantic and social systems. The present study investigated whether there is evidence of shared processing in domain-general regions or within a shared hub in ATL that support both systems at the individual and group level in the same naturalistic context.

For tractability, researchers tend to fractionate human cognition into modules and study these modules as independent, non-overlapping systems^7^. A prevalent, perhaps unintentional, example of this treatment of cognition can be seen in standard fMRI contrast analyses in which a cognitive process of interest is isolated by measuring an experimental condition (i.e., social communication) and subtracting from it a control condition that minimally requires the cognitive process of interest (i.e., non-social communication). Studies utilizing this methodology have generated significant insights into many aspects of human cognition, including both social and semantic cognition. This methodology assumes an additive relationship between the processes such that it can be undone by subtraction (i.e., control processes operate in the same way in the control and experimental conditions). Although this assumption may be approximately valid in many cases, it explicitly does not hold for integrated or interactive systems. Subtracting non-social communication from social communication to identify “social cognition” assumes that communication works the same way in social and non-social contexts (e.g., no social knowledge is nested within the subtracted semantic system, which conflicts with existing evidence^7^) and that social cognition works the same way in communication and non-communication contexts (i.e., subtracting the communication component leaves task-independent social cognition that would operate similarly in non-communication contexts). This research strategy has led to the impression that all cognitive systems are subtractable and independent in the mind and brain, rather than just being separate in the research literature.

In addition to this general treatment of human cognition, the relationship between these two cognitive systems has been obscured by differences in the types of paradigms used to study them. Studies have tended to rely on highly simplified experiments to investigate both semantic and social processing, but there is greater diversity in the content and presentation of paradigms used to study social cognition. Semantic tasks often involve single words or pictures, whereas the stimuli used in social tasks range from single word and sentence stimuli to face stimuli and social animations. As a result, the same social cognitive process (e.g., mentalizing) can be elicited by heterogeneous tasks (e.g., false-belief vignettes, comic strips, strategic games, animations) which complicates cross-task inferences due to varied task demands^42,43^. These methodological differences hinder investigations of shared processing between semantic and social cognition due to the challenge of identifying stimuli and paradigms with matched processing demands that meaningfully capture both systems. This would ideally be accomplished by eliciting semantic and social processing within the same paradigm.

Given the specific barriers that have hindered investigations of the interdependence between semantic and social cognition, it is critical to select stimuli that adequately capture varied social knowledge, including social semantic information (i.e., social concepts) and social interaction information (i.e., social events). An ideal avenue to accomplish these complementary goals is through the analysis of naturalistic neuroimaging data. Naturalistic neuroimaging provides greater ecological validity compared to studies of isolated word processing, which do not capture how the brain processes information in the real-world and disregards the context that informed the conceptual representation^44–46^. Social information occurs in dynamic and multimodal contexts in which knowledge accrues over several seconds to minutes, which naturalistic paradigms more closely approximate. One type of naturalistic paradigm, movie viewing, has been shown to produce stable intrinsic connectivity networks that are more reliable than those derived from resting state data^47,48^ and which provides the opportunity to capture temporal structure that would be lost in a traditional event-averaging fMRI analysis^49^. Further, humans segment continuous experiences into discrete events^50^ and cortical regions have varied temporal receptive windows that are directly impacted by the duration and content of these events^51,52^. Naturalistic paradigms allow for the investigation of both short (i.e., 1000 ms) and long temporal processing across cortical regions in response to varied content.

The aim of the present study was to investigate the shared and distinct neural organization of social, semantic, and semantic control brain networks by examining the response of these networks to semantic and social information in movies, while distinguishing between word-level and event-level representations. The study utilized the publicly available Naturalistic Neuroimaging Database (NNDb), which includes hours of movie viewing fMRI data for a large sample of adults (N = 86)^53^. Of note, the data include 10 different movies, which enables tests of generalizability of results across stimuli and provides an opportunity to sample varied social concepts and events. Independent ratings of semantic and social content from manually coded events for each movie were used as continuous event-level variables. Lexical and semantic content and smoothed factor scores indexing Semantic Flexibility and Social Impact were used as continuous word-level variables. The continuous variables were used to identify regions of the brain that respond to semantic and social content and to examine the degree that neural resources are shared between the systems at the individual and group level. The primary aims and predictions of the current study are divided into two complementary research questions (see Table 1).

First, during naturalistic movie viewing, is semantic, social, and semantically flexible (i.e., having several uses or meanings) content associated with increased activation in the semantic, social, and semantic control networks, respectively? It was expected that clusters of voxels showing increased activation in response to greater semantic, social, and semantically flexible word-level content will fall within the semantic (hypothesis 1.1a), social (hypothesis 1.1b), and semantic control (hypothesis 1.1c) brain networks, respectively. Further, it was expected that the clusters of voxels associated with semantic and social event-level content will fall within the semantic (hypothesis 1.2a) and social (hypothesis 1.2b) brain networks, respectively.

**Table 1:** Design table.

Question	Hypothesis	Analysis plan	Interpretation given to different outcomes
1. During naturalistic movie viewing, is semantic, social, and semantically flexible content associated with increased activation in the semantic, social, and semantic control networks, respectively?	1.1a (semantic words) Clusters of voxels showing increased activation in response to greater semantic word-level content will fall within the semantic brain network	The word-level analyses will be the same for semantic, social, and semantically flexible content. The following steps will be taken for each measure independently: (1) Extract smoothed time series of scores (either residual factor scores which account for number of words or number of content words) using a sliding window within event boundaries (2) Whole-brain parametric modulation analysis (3) The subject-level activation maps for a given content type will be used as inputs for a second-level group analysis using linear mixed-effects modelling with a fixed effect of content type and random intercepts of subject and movie (4) The statistical map will be corrected using a cluster-forming threshold of *p* < 0.01 and an FWE-corrected threshold of *p* < 0.05 (5) Results will be compared to the ALE-defined networks of interest, focusing on the core regions within each network, highlighted in the top panel of Fig. 1	Null: Fewer than 20 voxels will be associated with increased semantic information Alternatives: The clusters of voxels associated with increased semantic information will (1) include portions of the semantic network as well as regions outside the ALE-defined semantic network (partial support) or (2) fall entirely outside the ALE-defined semantic network (no support)
	1.1b (social words) Clusters of voxels showing increased activation in response to greater social word-level content will fall within the social brain network		Null: Fewer than 20 voxels will be associated with increased social information Alternatives: The clusters of voxels associated with increased social information will (1) include portions of the social network as well as regions outside the ALE-defined social network (partial support) or (2) fall entirely outside the ALE-defined social network (no support)
	1.1c (control words) Clusters of voxels showing increased activation in response to semantically flexible word-level content will fall within the semantic control brain network		Null: Fewer than 20 voxels will be associated with increased semantically flexible content Alternatives: The clusters of voxels associated with increased semantic flexibility will (1) include portions of the semantic control network as well as regions outside the ALE-defined semantic control network (partial support) or (2) fall entirely outside the ALE-defined semantic control network (no support)
	1.2a (semantic events) Clusters of voxels showing increased activation in response to semantic events (plot-progressing, informative verbal or written scenes) will fall within the semantic brain network	The event-level analyses will be the same for semantic, social, and scrambled content. The following steps will be taken for each measure independently: (1) Whole-brain duration modulated parametric analysis (2) The subject-level activation maps for a given content type will be used as inputs for a second-level group analysis using linear mixed-effects modelling with a fixed effect of content type and random intercepts of subject and movie (3) The statistical map will be corrected using a cluster-forming threshold of *p* < 0.01 and an FWE-corrected threshold of *p* < 0.05	Null: Fewer than 20 voxels will be associated with semantic events Alternatives: The clusters of voxels associated with increased semantic information within events will (1) include portions of the semantic network as well as regions outside the ALE-defined semantic network (partial support), (2) fall entirely outside the ALE-defined semantic network (no support), or (3) produce the same clusters as the scrambled ratings (no support)
	1.2b (social events) Clusters of voxels showing increased activation in response to social events (scenes depicting on or off-screen interactions between/among characters) will fall within the social brain network		Null: Fewer than 20 voxels will be associated with social events Alternatives: The clusters of voxels associated with increased social information within events will (1) include portions of the social network as well as regions outside the ALE-defined social network (partial support), (2) fall entirely outside the ALE-defined social network (no support), or (3) produce the same clusters as the scrambled ratings (no support)
2. To what extent are the semantic and semantic control networks involved in processing social concepts and events in individual subjects?	2.1a (semantic overlap) If there are clusters of voxels that respond to social word and event-level content, then it is expected that both social concepts and social events will engage areas of overlap within the semantic brain network defined within individual participants	The hypotheses of RQ2 will be tested using the following procedure: (1) The number of overlapping voxels will be calculated between the subject-level statistical maps for processing semantic and social content (word-level and event-level results processed independently) (2) The number of overlapping voxels will be calculated between the subject-level statistical maps for processing semantically flexible words and social content (word-level and event-level results processed independently) (3) A second-level random effects analysis will be run using the overlap images from individual participants to determine whether stable areas of overlap exist across participants	Null: At the individual subject level, fewer than 10 voxels show increased activation in response to both semantic content and social content (either concepts or events) Alternatives: (1) clusters of voxels will show increased activation in response to semantic content and social concepts, but not social events (partial support) or (2) clusters of voxels will show increased activation in response to semantic content and social events, but not social concepts (partial support)
	2.1b (control overlap) If there are clusters of voxels that respond to social word and event-level content, then it is expected that both social concepts and social events will engage areas of overlap within the semantic control brain network defined within individual participants		Null: At the individual subject level, fewer than 10 voxels show increased activation in response to both semantically flexible content and social content (either concepts or events) Alternatives: (1) clusters of voxels will show increased activation in response to semantically flexible content and social concepts, but not social events (partial support) or (2) clusters of voxels will show increased activation in response to semantically flexible and social events, but not social concepts (partial support)
	2.2a (semantic non-overlap) If there are clusters of voxels that respond to social word and event-level content, then it is expected that non-overlapping, proximal clusters of voxels will differentially respond to semantic and social content		Null: At the individual subject level, the voxels which respond to social content will not be proximal to the voxels which respond to semantic content (i.e., the clusters of voxels will not be sub-regions within a single atlas-defined anatomical region) Alternatives: (1) the voxels associated with processing social concepts, but not social events, will be proximal (i.e., sub-regions within a single anatomical region) to the voxels associated with processing semantic content (partial support) or (2) the voxels associated with processing social events, but not social concepts, will be proximal to the voxels associated with processing semantic content (partial support)
	2.2b (control non-overlap) If there are clusters of voxels that respond to social word and event-level content, it is expected that non-overlapping, proximal clusters of voxels will differentially respond to semantic control and social content		Null: At the individual subject level, the voxels which respond to social content will not be proximal to the voxels which respond to semantically flexible content (i.e., the clusters of voxels will not be sub-regions within a single anatomical region) Alternatives: (1) the voxels associated with processing social concepts, but not social events, will be proximal (i.e., sub-regions within a single anatomical region) to the voxels associated with processing semantically flexible content (partial support) or (2) the voxels associated with processing social events, but not social concepts, will be proximal to the voxels associated with processing semantically flexible content (partial support)

The sampling plan is the same for all tested predictions. A sensitivity power analysis was conducted using using the *pwr* package in R. With the fixed sample size of 86, statistical power of 0.95, and an alpha of 0.05, an omnibus multiple regression analysis with 2 to 3 predictors would be sensitive to detecting medium effects (*f*^2^ = 0.19–0.21).

Second, to what extent are the semantic and semantic control networks involved in processing social concepts and events in individual subjects? If there are clusters of voxels that respond to social word-level and event-level content, then it was expected that both social concepts and social events will engage areas of overlap within the semantic (hypothesis 2.1a) and semantic control (hypothesis 2.1b) brain networks defined within individual participants. This would provide evidence of shared resources between the social and semantic systems. If that overlap occurs within the known semantic hub, ATL, this will provide support for the *graded semantic hub* hypothesis, suggesting that the systems leverage a shared hub. In addition to overlap, it was expected that non-overlapping, proximal clusters of voxels will differentially respond to semantic and social content (hypothesis 2.2a) and semantic control and social content (hypothesis 2.2b), providing evidence of graded functioning within network regions.

## Materials

### Ethics information

The research complies with all relevant ethical regulations. The project from which the data are derived was approved by the ethics committee of University College London. Participants provided written informed consent to take part in the study and have their anonymised data shared.

### Design

The research questions were tested via secondary analyses of a publicly available dataset called the Naturalistic Neuroimaging Database (NNDb) which is accessible on OpenNeuro (https://openneuro.org/datasets/ds002837/versions/2.0.0). Version 2.0.0 of the database (released April 20, 2021) was used for all analyses and includes the raw and preprocessed data from 86 participants (mean age = 26.81; 42 females) who watched one of ten movies (length range = 5470—8882s) while undergoing fMRI. Movie selection was decided by previous exposure, so all participants were shown a movie they had not previously watched. At least 6 and up to 20 participants watched each movie.

None of the authors had previously analysed the participant data from any version of this dataset nor had any direct knowledge of the data at the time of pre-registration. All analyses were registered prior to any human observation of the neuroimaging data. The movie annotation files were obtained prior to registration and coded using protocols designed to (1) segment the movies into discrete events and (2) derive a range of continuous variables encoding the presence of word and event-level semantic and social information at each point in time (see below). For a detailed overview of the experimental procedures, including how the data were collected and preprocessed, see the publication describing the dataset^53^.

### Sampling plan

The current study is a secondary analysis of existing data, and, as such, the sample size is fixed. With 86 participants who each watched a full feature-length film, the NNDb is among the largest publicly available databases of naturalistic neuroimaging data to date (although see the *Narratives* dataset^54^), and currently the largest dataset that uses movie stimuli. Comparable databases often have fewer than 30 participants, and many use stimuli that are less coherent (i.e., clips of adverts or scenes from films) or shorter in duration (i.e., single episodes or short films). Task-based fMRI studies require more than 80 participants to detect medium effect sizes (see power analysis below)^55^, and scan times greater than 90 min produce more reliable results^56^. With the large sample size and the longer scan duration, the NNDb provides more data per participant than many other naturalistic neuroimaging databases.

A sensitivity power analysis was conducted using the *pwr* package in R^57^ to determine what effect size is detectable given the fixed sample size. This type of power analysis is a complement to the more common a priori power analysis which assumes an effect size and computes the sample size necessary to detect that effect. With the fixed sample size of 86, statistical power of 0.95, and an alpha of 0.05, an omnibus multiple regression analysis with 2–3 predictors would be sensitive to detecting medium effects (*f*^*2*^ = 0.19–0.21). This is a conservative estimate of statistical power because it does not take into consideration the large number of observations (i.e., time points) within participants which substantially increases statistical power, especially when within participant variance in the dependent variable is high. Similar effect sizes are detectable in standard event-related and blocked design fMRI experiments but require many trials (k > 60) or a larger sample (N > 30)^58^, both of which are far exceeded with the NNDb dataset.

### Analysis plan

**Figure 2:**
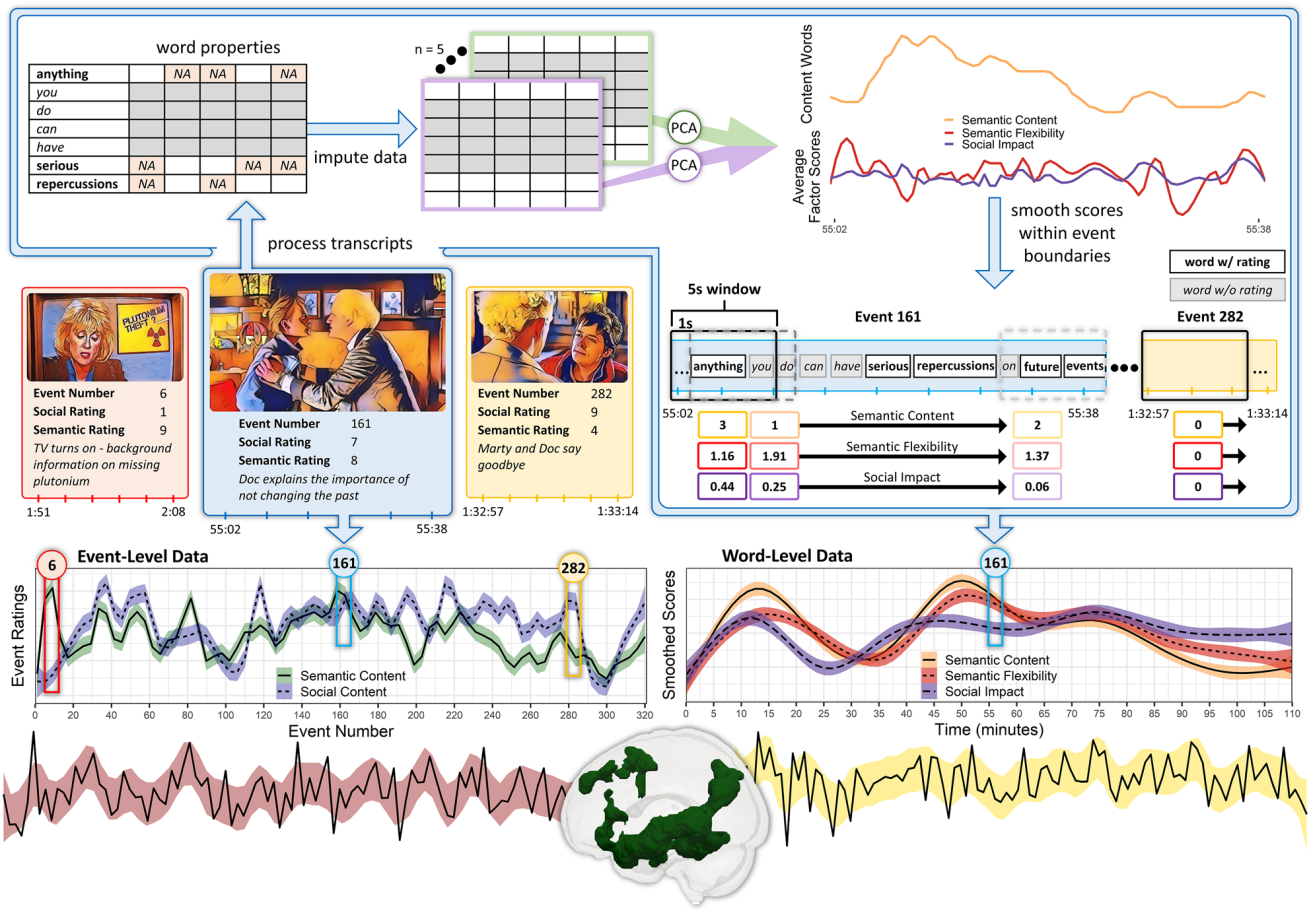
Schematic of analyses. Representative events from one of the movies (*Back to the Future*) are shown in red, blue, and yellow shaded tiles. The corresponding event number, social and semantic rating, and event description are provided below the event screenshot. These events are sampled from the Event-Level time course (below the tiles) which shows the semantic (green) and social (blue) event ratings for the movie. Event 161 (blue tile) is used to illustrate how the words within events are processed. First, word properties are generated for all transcript words. Second, missing data are imputed 5 times (ignoring closed class words which are shaded grey). Third, PCA is run on each of the imputed datasets, and the resulting factor scores from the 5 datasets are averaged. Only the averaged Semantic Flexibility and Social Impact factor scores are shown because these are the data used for analyses. Fourth, the factor scores and semantic content (i.e., number of content words) are smoothed within the event boundaries. The summed total factor score or the number of content words is calculated within a 5s sliding window sliding every second. This window stops at the end of the event, and a new sliding window starts at the beginning of each event. When no words fall within a window (demonstrated with Event 282), the calculated window value is 0. This process results in a Word-Level time course of smoothed scores which is shown to the right of the Event-Level time course. Both the Event-Level and Word-Level time courses were used as parametric modulators by convolving the time courses with whole-brain BOLD signal (bottom panel). The semantic network and simulated BOLD time series are shown as an example.

In order to investigate neural processing of social and semantic events and concepts, two primary types of scores were extracted from each movie: (1) event-level scores and (2) word-level scores. The movie event-level scores were generated via a manual coding process in which each movie was segmented into discrete events and the relative semantic and social content within events were rated independently. Movie word-level scores were generated by conducting a principal component analysis on 12 critical word property values and smoothing the resulting scores using a sliding window. Both the word-level and event-level scores were used in parametric modulation analyses to assess the neural response to varied levels of lexical and event-based semantic and social content (RQ1) and the extent to which processing lexical and event-based semantic and social information relies on overlapping regions within the semantic and social brain networks within subjects (RQ2). The sections below provide additional detail on how these data were generated and the pre-registered analyses. See Fig. 2 for a schematic overview of the analyses.

#### Movie events

To provide data at the event level, each movie was segmented into discrete events capturing subtle changes in the content or purpose of consecutive scenes. A detailed protocol was developed to provide consistent principles for segmenting. Manual subjective ratings of event boundaries have been previously applied to naturalistic movie data and have a high degree of correspondence with data-driven event segmentation models based on shifts in patterns of brain activity^50^. For this study, event transitions were identified by the first author using the following criteria: (1) event boundaries are defined by a qualitative shift in the tone, setting, characters, or purpose of the scene; (2) a single event is often shot continuously with the same characters in the same setting or environment. Any sudden shift in the tone or emotional impact should be an indicator that a new event has begun; (3) the action sequences that occur within an event are more predictable than action sequences that occur between events. The latter criterion was derived from previous research on event prediction, which suggests that a good indicator of whether a new event has begun is if a sequence feels disconnected, unexpected, unrelated, or discontinuous from the previous sequence^59,60^. Changes in background music or camera angles alone were not sufficient for marking an event boundary. Further, a distinction was made between *major events*, which tend to occur less frequently, have a longer duration (i.e., several minutes), and be accompanied by a larger shift in the content or purpose of a scene, and *minor events*, which occur more frequently, have a shorter duration (i.e., several seconds), and are signalled by more subtle shifts in content or purpose. Excluding opening studio credits and closing credits, the number of events per movie ranged from 238 to 429 (*Mdn* = 384).

The semantic and social content of each minor event was rated for each movie. Both semantic and social content were rated on a scale from 1 to 10 with higher scores indicating greater semantic or social content. Semantic content was defined as narrative exposition in which movie or scene information is presented linguistically through spoken language (by a character or narrator) or in writing (such as text about the movie, timescale, or characters or any text presented during an event). Although semantic information can be expressed non-linguistically, this type of semantic knowledge requires a greater degree of inference which can be variable when manually coding events. To avoid conflating linguistic semantic information with non-linguistic semantic or pragmatic inferences, only spoken or written information was considered semantic content. For the purposes of this study, events in which new semantic information was presented were coded as more semantic relative to events with semantic content that was already known to the viewer. Critically, a distinction was made between novel information and shocking or surprising information, the latter of which did not receive a higher semantic score. Information was considered new only if it is being presented to the viewer for the first time. Events in which a character learns information the audience already knows would receive a lower semantic score. Such a scene may receive a higher social rating (described below) if the information is personally impactful or requires updating a false belief. This criterion was included because events with novel information are more informative and require greater semantic processing relative to the moments in which the information is consistent or has already been processed (because it has already been presented to the viewer). Low semantic events would have minimal to no written or spoken exchange of new information, such as an action sequence. See Supplementary Fig. S1 for the detailed rubric for coding semantic information.

Social content was defined by the presence of more than one person or character, even if inanimate or off-screen. Any event that conveys information about the characters in the movie and their relationships with other characters was considered social. This could include general conversation or exchange of neutral character information, which would receive a low to moderate social rating, or could convey character attitudes, thoughts, feelings, or passions, which would receive a moderate to high social rating. The relative degree of sociality may depend on the type, duration, and significance of the interaction within the event. Events were considered more social if (1) the interpersonal connection between or among the characters was deeper and intense rather than superficial or brief based on their prior interactions throughout the movie and (2) the specific characters in the event bring a larger significance to the interaction based on who they are or the pre-defined relationship between or among the characters. A distinction was made between high social and high emotional content. Although an event may be both highly social and emotional, an event does not have to be emotionally intense in order to be considered social. Similarly, social and semantic content were coded independently as an event can be both highly (or weakly) social and semantic. The highest ratings (i.e., 9 or 10) were reserved for events in which the primary purpose of the scene was to convey semantic or social information. Importantly, a single event could not receive a 9 or 10 for *both* semantic and social content because the primary purpose had to be coded as either semantic or social. See Supplementary Fig. S2 for the detailed rubric for coding social information.

The first author watched all of the movies and marked the minor event boundaries using the established protocol. This was done to provide the event boundaries for coding semantic and social content and to ensure that the events of primary interest were coded in a consistent way across all 10 movies. At least 1 other independent coder then marked where the major events occurred within each movie using the established timestamps from the minor events.

After events were delineated, at least two independent coders (the first author and at least one independent coder) rated the semantic and social content of each minor event in the movie and wrote a brief description of what occurred during the event (see Table 2 for examples). Inter-rater reliability was assessed separately for the semantic and social scores for the coders of each movie using Krippendorff’s alpha reliability coefficient. When the inter-rater reliability fell below 0.75, the coder who rated all movies identified which events were poorly aligned, rewatched the event, and made a revised consensus rating based on the content of the event and the notes of the other coder.

**Table 2:** Example movie event annotations and smoothed word data.

Event data				
Event number	Semantic score	Social score	Event notes	
	R1 (R2)			
278	2 (3)	1 (1)	Doc waiting for Marty at the clock tower	
279	5 (6)	6 (6)	Marty arrives and Doc rushes over	
280	6 (6)	6 (6)	Marty explains how things went down with his dad. Doc seems worried	
281	9 (9)	4 (6)	Doc explains plan to Marty	
282	4 (5)	9 (10)	Marty and Doc say goodbye	
283	7 (7)	6 (4)	Doc restates part of the plan + Marty gets in the car	
284	7 (9)	5 (3)	Doc discovers letter in his coat + rips it up	
285	3 (4)	4 (5)	Tree crashes down. Doc and Marty split up to fix cables	
286	1 (3)	3 (5)	Doc runs up clock tower to throw cable over	
287	7 (7)	6 (7)	Marty tries to tell Doc about the future again	

Word data				
	Word Length	Semantic Flexibility	Emotional Strength*	Social Impact
Positive	[Event 170]: *…felt sorry for him cause her dad hit him with the car he hit me with…*	[Event 191]: …*know what to say say anything say whatever’s natural the first thing that comes into your mind…*	[Event 23]: *…Dr Brown is dangerous he’s a real nutcase hang around with him you’ll end up in big trouble…*	[Event 221]: *…I wish I wasn’t so scared there’s nothing to be scared of all it takes is self-confidence…*
Negative	[Event 281]: *…I’ve calculated the precise distance taking into account the acceleration…*	[Event 151]: *…the sink that’s when you got the idea for the flux capacitor which…*	[Event 81]: *…this tells you where you’re going this where you are and this where…*	[Event 38]: *…replace that clock thirty years ago lightning struck that clock tower and the clock…*

Event Data: representative movie event annotations are taken from *Back to the Future*. Event 282 is used as an example in Fig. 2. For the Semantic Score and Social Score ratings, the primary coder ratings (R1) are shown as well as ratings from a secondary coder (R2) which are shown in parentheses. Event Notes are from the primary coder (R1). Event Number refers to the Minor Event Number. Word Data: smoothed windows with high positive or negative summed total factor scores from *Back to the Future*. The event number from which each smoothed window is taken is indicated in brackets. *using absolute value transformed scores.

#### Movie transcripts

To provide data at the word level, the transcript annotations made available with the public dataset were used and included the words that were spoken as well as their onset and duration times. The initial paper describing the NNDb provides greater detail on the methods used for generating this information^53^. The following word properties were obtained, where available, for each word in the transcript annotations using the English Lexicon Project database^61^: number of letters, number of phonemes, number of phonological neighbours, number of orthographic neighbours, word frequency, concreteness^62^, semantic neighbourhood density, semantic diversity^63^, emotional valence (i.e., pleasantness), emotional arousal (i.e., intensity), and emotional dominance (i.e., control)^64^, and part of speech.

To obtain ratings of socialness for each word spoken in the movies, social word ratings were generated from a previous norming study conducted with 68 participants from the University of Alabama at Birmingham. Candidate words were derived from the Glasgow norms study, which includes normative psycholinguistic ratings for over 5000 individual words^65^. This initial list was filtered to remove words with high concreteness (> 5) and imageability (> 5) ratings in order to identify possible social concepts (which tend to be abstract, although see^15^) as norming targets. Additional target words were added from a study reporting social desirability ratings on over 500 words^66^. A randomly selected subset of 688 words were included in the norming study, and words with varied parts of speech were intentionally retained. During the norming study, participants were instructed that a word is social if it describes inter-personal behaviours, motivations, intentions, or characteristics and were asked to rate how social each presented word was on a scale from 1 (not social) to 5 (very social). Each participant rated half of the words resulting in 34 ratings for each of the 688 unique words. For words not present in this set of 688 words, social ratings were extrapolated by calculating their semantic similarity to each of the words in the normed set. Semantic vectors were generated for each of the normed words as well as for the unique transcript words using word2vec. The cosine similarity between each transcript word and every normed word was calculated resulting in 688 similarity values for each transcript word. The average social rating was then calculated by taking a weighted average, using the cosine similarity, of the social scores from the 10 closest semantic neighbours.

**Figure 3:**
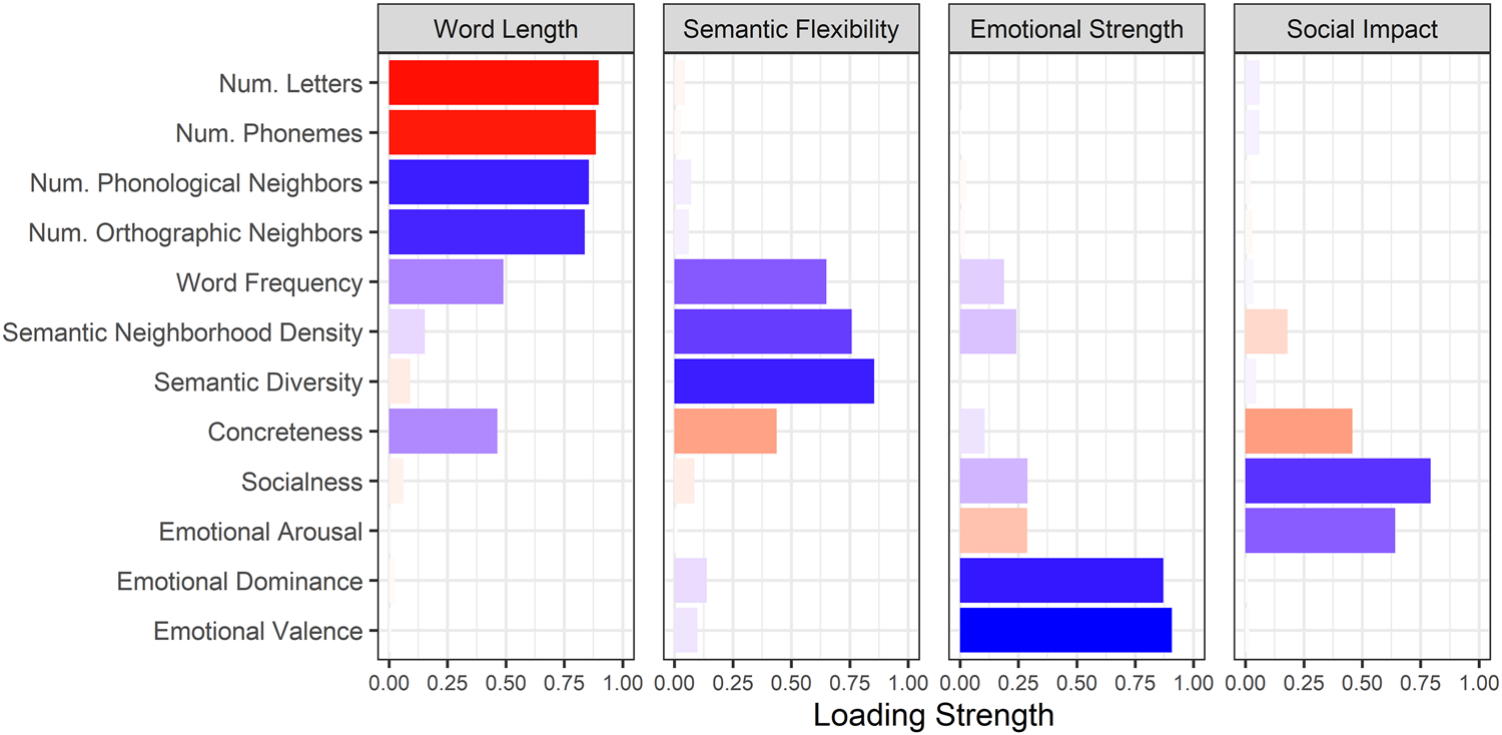
Factors derived from PCA on word property values. Positive (blue) and negative (red) loadings are shown for each factor. The strength of the loading is indicated by the length and colour saturation of each bar. Num., Number.

Prior to subsequent analyses, the unique words from all movies were combined; high frequency and closed class words were excluded, as were any words missing more than 10 of the 12 critical word properties (excluding part of speech). To avoid listwise deletion, missing data for the remaining set of words were imputed. Imputation was performed using the multiple imputation by chained equations approach implemented with the *mice* package in R^67^, and resulted in 5 complete datasets. To reduce covariance between predictors, the 12 word property measures for each unique word were entered into a principal component analysis (PCA) with varimax rotation for each imputed dataset. The four factor result corresponded to Word Length (e.g., number of letters and phonemes, number of phonological and orthographic neighbours), Semantic Flexibility (e.g., semantic diversity, semantic neighbourhood density), Emotional Strength (e.g., emotional valence, emotional dominance), and Social Impact (e.g., socialness, emotional arousal) and accounted for approximately 29%, 17%, 16%, and 11% of the variance respectively (Fig. 3). The Emotional Strength factor scores were transformed by taking the absolute value in order to capture emotional versus neutral content rather than positively versus negatively valenced content. The resulting factors were stable across the imputed datasets and resulted in the same factors as a PCA run on a subset of the data with no missing values. Due to random variation introduced when imputing data and given the robustness of the results, the factor scores derived from the imputed datasets were averaged.

#### Aligning annotations and events

The transcript annotations were temporally aligned with the events using the event boundary timestamps and the onset times of each word. Different versions of the movie files may have slightly varying playback speeds (± 1.5%). To ensure correct alignment, the events were marked and aligned using the same movie files that were presented to participants in the NNDb study. This was done to identify which words were present within each event. To account for hemodynamic lag, a smoothed time course of critical word-level factor scores was generated by summing values within a 5 s window sliding every 1 s. A data-driven event segmentation approach with comparable movie data found that the median duration of neural states across voxels ranged from 5 to 18.5 s, and these durations were reliable across participants^68^. A 5 s window is thus advantageous as it would capture the regions with shorter neural state durations (predominately sensory processing areas) and provide multiple measurements of the neural state of those regions with longer durations (e.g., default mode network). Importantly, the sliding windows were constrained to each event’s boundaries so word property scores from different events were not averaged together (see Table 2 for examples). Events with excessively short duration (< 3 s; 4% of events) were merged with the subsequent event by taking the average semantic and social score across the two events. After merging short events, the event duration ranged from 4 to 131 s (*Mdn* = 16.00 s). If an event was shorter than the window size of 5s, the sum was calculated within this smaller window. If the final portion of an event was less than 1 s (i.e., 500 ms), a slightly shorter window was defined, and the sum was calculated within the smaller window. For each factor, the residuals were extracted from a model predicting the smoothed factor time course from the number of total words within each window. This controlled for the number of words in an event. If there are no words or no words that have ratings within a window or event, a value of 0 was assigned for each factor score.

General lexical-semantic content was quantified by counting the number of content words (i.e., open class words). Open class words with missing word property data (n = 883) which were excluded from the PCA (and subsequently do not have factor scores) were still counted as semantic content by manually tagging part of speech. These include character names, dates, or movie-specific words (i.e., fictional towns, technology, slang) that were not found in psycholinguistic databases. The same sliding window approach was used to generate a smoothed time course of lexical-semantic content. This predictor, as well as the factor scores, captures the momentary quantity of basic conceptual knowledge within each window, agnostic to the preceding events, which likely under approximates the amount of semantic processing occurring and is not sensitive to detecting pragmatic inference or non-linguistic semantic processing. This approach was adopted here because it most closely aligns with how prior studies examined semantic processing of isolated words or sentences, and one of the goals of the current study was to examine these measures in a naturalistic context. The event-level semantic predictor similarly indexes only the linguistic information in events, but is informed by prior context and may better capture broader context-sensitive semantic processing.

#### Network definitions

The networks of interest were defined using statistical maps derived from independent coordinate-based activation likelihood estimation (ALE) analyses of semantic control, semantic cognition, and social cognition and are shown in Fig. 1. These network maps were used to aid in the interpretation of the results of the whole brain analyses by categorizing results as falling within or outside of the pre-defined networks. The semantic and social networks in particular are extensive, and it is likely to be minimally informative to look at percent overlap or Dice similarity coefficient in isolation. For this reason, greater emphasis was placed on *where* overlap between the resulting networks and the pre-defined networks occurs. Overlap in core regions within each ALE-generated network, highlighted in the top portion of Fig. 1, was interpreted as stronger evidence of network involvement. Defining networks prior to analysis ensured that results were interpreted in a pre-specified manner.

The semantic control network was defined based on an ALE analyses with over 120 contrasts from 87 studies which identified a large cluster centred around the left inferior frontal gyrus as well as posterior middle temporal gyrus, dorsomedial prefrontal cortex, and a smaller portion of the right inferior frontal gyrus^39^. This network has significant convergence with another ALE-generated semantic control network^31^. The same study also identified a general semantic cognition network derived from over 400 contrasts from 257 studies. In order to isolate the automatic semantic network and partial out the role of control in semantic retrieval, the semantic control ALE result was subtracted from the general semantic cognition ALE result and small clusters of voxels (< 400 contiguous voxels) were removed. This resulted in a map which included left anterior temporal lobe, left medial and posterior temporal cortex, left inferior parietal lobule, insula, precentral gyrus, and right middle and superior temporal gyrus.

The social cognition network was defined by examining the convergence of ALE generated network maps for four primary domains of social cognition from a previous study^31^. These domains included theory of mind (derived from 136 experiments), trait inference (derived from 40 experiments), empathy for pain or affective states (derived from 163 experiments), and moral reasoning (derived from 68 experiments). The ALE results for each domain were overlaid and regions which were identified in at least one of the four domains were retained. This resulted in a social network which included bilateral inferior frontal gyrus, superior frontal gyrus, medial prefrontal cortex, precuneus, bilateral inferior parietal lobule, supplementary motor area, bilateral anterior and superior middle temporal gyrus, bilateral anterior temporal poles, and precentral gyrus.

### Statistical analyses

The pre-processed MRI data were used for all analyses. The pre-processing steps are documented in the initial paper describing the NNDb dataset^53^. The functional runs were concatenated into a single timeseries file after detrending and censoring TRs with excessive motion. There are no missing data for the current set of 86 participants that comprise the NNDb. The only data that are missing are word property values, but the approach to dealing with those data are outlined in detail above. The NNDb data includes quality assurance metrics indexing movement related artefacts and signal to noise. Given that the metrics indicate that the data are high quality (mean temporal signal to noise ratio [tSNR] > 60) and the scan duration was sufficiently long for detecting activation at that tSNR level (> 1 h)^69^, none of the participants were excluded from analysis.

#### Research question 1

For the word-level analyses, the Semantic Flexibility factor was used in the semantic control analysis because semantic diversity^63^ and semantic neighbourhood density^70,71^ reflect increased selection demands which requires additional cognitive control. The Social Impact factor was used as a proxy of social content, and the number of content words was used as a coarse measure of semantic content. These measures were each separately used as parametric modulators in a regression model predicting the time series from the full movie. The analyses were conducted at the whole-brain level, and results were compared to the predefined networks of interest. The subject-level activation maps for each content type for all movies were used as inputs for a second-level group analysis using linear mixed-effects modelling with a fixed effect of content type (i.e., social, semantic, or semantically flexible) and random intercepts of subject and movie implemented using 3dLMEr in AFNI^72^. The resulting statistical map were corrected using a cluster-forming threshold of *p* < 0.01 and an FWE-corrected threshold of *p* < 0.05.

For the event-level analyses, the semantic and social event ratings for each movie were used in a first-level general linear model for each subject as parametric modulators predicting the time series from the full movie. To account for the varied event durations, a duration modulated model was used in which the onset and duration of each event were included in the regression model. Either semantic or social content were included as a nuisance regressor. In addition, the ratings were randomly scrambled to generate a null distribution which served as a comparison condition with no semantic or social content. The analyses were conducted at the whole-brain level. The subject-level activation maps across all movies were used as inputs for a second-level group analysis using linear mixed-effects modelling with a fixed effect of content type (i.e., social or semantic or null) and random intercepts of subject and movie implemented using 3dLMEr in AFNI^72^. The resulting statistical map was corrected using corrected using a cluster-forming threshold of *p* < 0.01 and an FWE-corrected threshold of *p* < 0.05.

#### Research question 2

To investigate the extent to which the social system shares neural resources with the semantic and semantic control systems within individual subjects, the subject-level statistical maps generated to test research question 1 were directly compared. Specifically, the number of overlapping voxels was calculated between (1) the statistical maps for processing semantic and social content (word-level and event-level results processed independently) and (2) the statistical maps for processing semantically flexible words and social words. The presence of voxels that respond to *both* semantic and social concepts or events was taken as evidence of shared neural resources between the systems. The strength of the evidence was determined by the number of overlapping voxels quantified using Dice coefficient, and fewer than 10 overlapping voxels was considered functionally the same as 0 voxels. Overlap within the semantic hub in ATL will provide support for the *graded semantic hub hypothesis* which suggests that both systems rely on the same domain-general hub. Non-overlapping, proximal clusters of voxels that differentially respond to semantic (or semantic control) and social content in the absence of any overlapping voxels will provide weaker evidence of shared processing between the systems, and instead will be interpreted as evidence for graded functioning within a semantic or social network region. To determine the extent to which stable areas of overlap between the cognitive systems exist across participants, a second-level random effects analysis was run using the overlap images from individual participants. The group-level overlap maps were compared to the predefined ALE-derived network definitions to determine the extent to which the core regions within each network (shown in Fig. 1) were involved in each process and to isolate any regions which fall outside the expected networks.

## Results

### Research Question 1

The Words and Events results are shown in the following sections with two cluster correction thresholds applied: (1) the pre-registered threshold (cluster-forming threshold of *p* < 0.01 with an FWE-corrected threshold of *p* < 0.05) and (2) a more conservative threshold (cluster-forming threshold of *p* < 0.01 with an FWE-corrected threshold of *p* < 0.01). The latter threshold was applied in an effort to highlight areas with the strongest response to the stimulus alongside the values that fall below that threshold^73^. The results figures indicate which voxels survived each cluster threshold and tables report clusters that survived the pre-registered threshold. Results figures were generated using MRIcroGL^74^ and the *ggplot2*^75^, *ggdist*^76^, and *gghalves*^77^ packages in R^78^.

#### Words analyses

**Figure 4:**
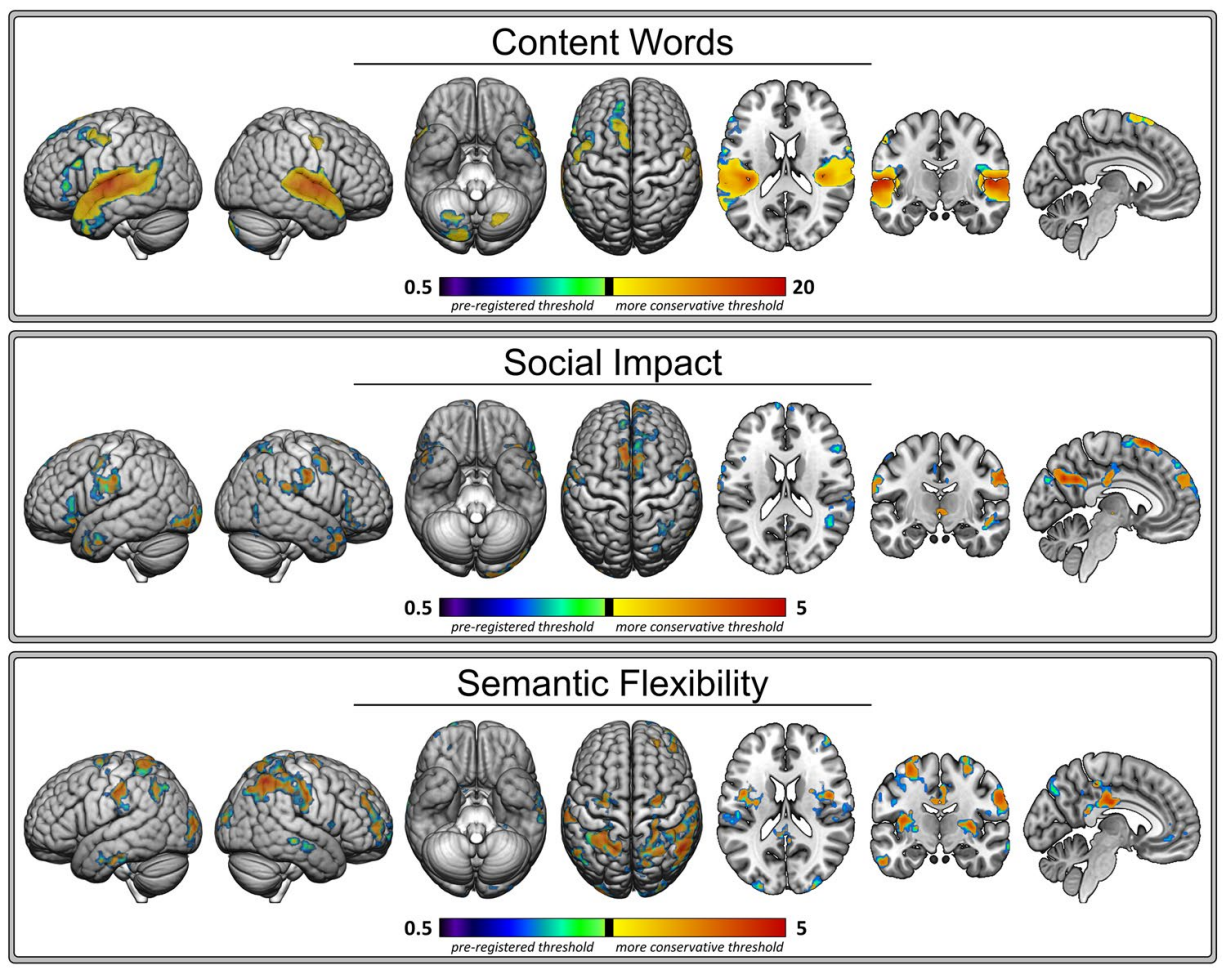
Words Analyses Results. Thresholded Z-score statistical maps showing the number of content words (top panel), Social Impact (middle panel), and Semantic Flexibility (bottom panel) results. All clusters survived the pre-registered cluster threshold. The clusters that survived an additional, more conservative threshold are indicated in yellow (lower) to red (higher). The clusters that did not survive the more conservative threshold are shown in purple (lower) to green (higher).

The word-level predictor variables were generated as described in the pre-registered Methods section with one minor deviation. The pre-registration indicated that when a window or event contained no words or no words that had ratings, a value of 0 would be assigned for each factor score. However, because scores were mean-centred, a score of 0 corresponds to words with an average factor score, not the absence of a score as initially intended. In addition, given the visual nature of movie stimuli there are many events that do not contain words. As a result, many windows containing few, if any, words would have been modelled as containing words with average factor scores. This was not justifiable on scientific grounds, so windows with no words or no words with ratings were removed from analysis instead. This error was realized and corrected prior to running the words analyses. The words analysis results are shown in Fig. 4 and the coordinate information is provided in Table 3.

**Table 3:** Words results coordinate table.

Variable	Cluster size	Hem	Brain region peak voxel	Brain region highest overlap [%]	MNI coordinates		
					X	Y	Z
Content words	2558	L	Superior Temporal Gyrus	Middle Temporal Gyrus [30%]	− 53	− 20	7
	1689	R	Heschl’s Gyrus	Superior Temporal Gyrus [42%]	50	− 14	7
	418	R	Cerebellum (VII)	Cerebellum (Crus 2) [32%]	17	− 83	− 57
	175	L	Supplementary Motor Area	Superior Frontal Gyrus [41%]	− 5	4	77
	149	L	Postcentral Gyrus	Precentral Gyrus [29%]	− 56	− 10	57
	117	L	Inferior Frontal Gyrus (Triangularis)	Inferior Frontal Gyrus (Triangularis) [76%]	− 56	16	29
Semantic Flexibility	2423	R	Inferior Parietal Lobule	Postcentral Gyrus [17%]	53	− 57	51
	802	L	Superior Parietal Lobule	Postcentral Gyrus [27%]	− 17	− 64	73
	471	R	Putamen	Putamen [32%]	32	4	5
	375	R	Middle Frontal Gyrus	Middle Frontal Gyrus [58%]	41	51	8
	331	L	Insula Lobe	Insula Lobe [35%]	− 32	1	5
	284	R	Middle Orbital Gyrus	Middle Orbital Gyrus [19%]	8	39	− 7
	259	L	Cerebellum (VI)	Fusiform Gyrus [39%]	− 26	− 43	− 33
	220	L	Inferior Occipital Gyrus	Middle Occipital Gyrus [84%]	− 26	− 91	− 3
	169	L	Precentral Gyrus	Precentral Gyrus [55%]	− 29	− 17	66
	168	R	Middle Occipital Gyrus	Middle Occipital Gyrus [53%]	29	− 95	10
	116	R	Superior Frontal Gyrus	Superior Frontal Gyrus [83%]	26	− 11	76
	110	L	Angular Gyrus	Angular Gyrus [35%]	− 53	− 66	44
	99	L	Inferior Temporal Gyrus	Inferior Temporal Gyrus [67%]	− 56	− 12	− 38
	99	R	Middle Temporal Gyrus	Middle Temporal Gyrus [91%]	65	− 29	− 14
Social Impact	576	L	Precuneus	Precuneus [30%]	− 5	− 68	31
	463	L	Inferior Frontal Gyrus (Orbitalis)	Inferior Frontal Gyrus (Orbitalis) [24%]	− 32	18	− 19
	383	R	Inferior Frontal Gyrus (Orbitalis)	Inferior Frontal Gyrus (Orbitalis) [25%]	38	24	− 18
	367	L	Postcentral Gyrus	Postcentral Gyrus [43%]	− 71	− 15	24
	341	L	Supplementary Motor Area	Supplementary Motor Area [44%]	− 8	20	74
	335	R	Superior Medial Gyrus	Superior Medial Gyrus [43%]	5	62	31
	300	R	Postcentral Gyrus	Supramarginal Gyrus [40%]	62	− 16	40
	252	R	Superior Temporal Gyrus	Superior Temporal Gyrus [43%]	44	− 29	− 7
	222	L	Lingual Gyrus	Inferior Occipital Gyrus [32%]	− 17	− 103	− 15
	162	R	Superior Parietal Lobule	Superior Parietal Lobule [58%]	35	− 57	57
	159	R	Angular Gyrus	Angular Gyrus [64%]	50	− 59	28
	138	R	Cerebellum (Crus 1)	Inferior Temporal Gyrus [38%]	47	− 62	− 27
	115	R	Middle Cingulate Cortex	Middle Cingulate Cortex [32%]	− 2	− 24	26
	111	R	Precentral Gyrus	Precentral Gyrus [79%]	50	− 7	57

This table was generated based on the pre-registered cluster threshold. Cluster size is determined by the number of 2 mm^3^ voxels. MNI coordinates correspond to the voxel with peak activation within each cluster. Voxels were defined as neighbours based on faces touching (NN = 1). Atlas labels are based on the Eickhoff-Zilles macro labels from the N27 (MNI space) atlas. Hem, Hemisphere; L, Left; R, Right.

##### Number of content words

It was expected that clusters of voxels showing increased activation in response to greater semantic word-level content (i.e., number of content words) would fall within the semantic brain network (Hypothesis 1.1a). In line with this prediction, the Dice similarity coefficient was higher for this result and the semantic network (0.25) than either of the other word-level content results (0.01–0.06; see red diamonds in Fig. 7). An increase in the number of content words was positively associated with activation in broad bilateral clusters extending from anterior to posterior superior temporal gyri with peak voxels in auditory cortices. The left hemisphere cluster was more extensive, including the superior and lateral portions of the temporal pole (the lateral portion is sometimes labelled “ventrolateral ATL”^24,27^, though the present results did not extend to the ventral portion of ATL), middle temporal, supramarginal, and angular gyri. Frontal activation was observed in smaller clusters in left inferior frontal, middle frontal, and superior frontal gyri and supplementary motor area and left precentral gyri. Cerebellar activation, predominately in the right posterior lobe of the cerebellum, also positively co-varied with the number of content words.

##### Social Impact

It was expected that clusters of voxels showing increased activation in response to greater social word-level content (i.e., Social Impact scores) would fall within the social brain network (Hypothesis 1.1b). In line with this prediction, the Dice similarity coefficient was higher for this result and the social network (0.20) than either of the other word-level content results (0.01–0.05; see dark blue diamonds in Fig. 7). An increase in social and emotionally arousing words (indicated by positive Social Impact scores) was associated with activation in precuneus, right inferior parietal lobule (i.e., temporo-parietal junction [TPJ]), and frontal activation in bilateral inferior frontal gyri, superior medial gyrus, supplementary motor area, right precentral and middle frontal gyri, and left postcentral gyri. Activation in bilateral anterior middle (i.e., ventrolateral ATL) and superior (i.e., dorsolateral ATL) portions of the temporal pole also positively co-varied with Social Impact, as did clusters in right inferior temporal gyrus and fusiform and left inferior occipital gyrus.

##### Semantic Flexibility

It was expected that clusters of voxels showing increased activation in response to semantically flexible word-level content would fall within the semantic control brain network (Hypothesis 1.1c). Counter to this prediction, the Dice similarity coefficient was lower for this result and the semantic control network (0.00) than either of the other word-level content results (0.06–0.07; see orange diamonds in Fig. 7), although overlap was minimal across all word analysis results. Activation in left IFG and pMTG did not positively co-vary with Semantic Flexibility. Instead an increase in more frequent, semantically diverse words (indicated by positive Semantic Flexibility scores) was associated with activation in a large cluster with a peak voxel in right postcentral gyrus that included portions of middle cingulate cortex, inferior and superior parietal lobule, and precuneus. Activation in a smaller, analogous left hemisphere region positively co-varied with Semantic Flexibility as did clusters in anterior cingulate, right middle and superior frontal gyri, left precentral gyrus, bilateral insula, left inferior temporal and fusiform gyri, left angular gyrus, and bilateral middle occipital gyrus. These results suggest that processing Semantic Flexibile words in a movie context does not elicit semantic control processes as expected. This undermines its use in isolating the semantic control network within individual participants, an analysis planned to address Research Question 2.

#### Events analyses

**Figure 5:**
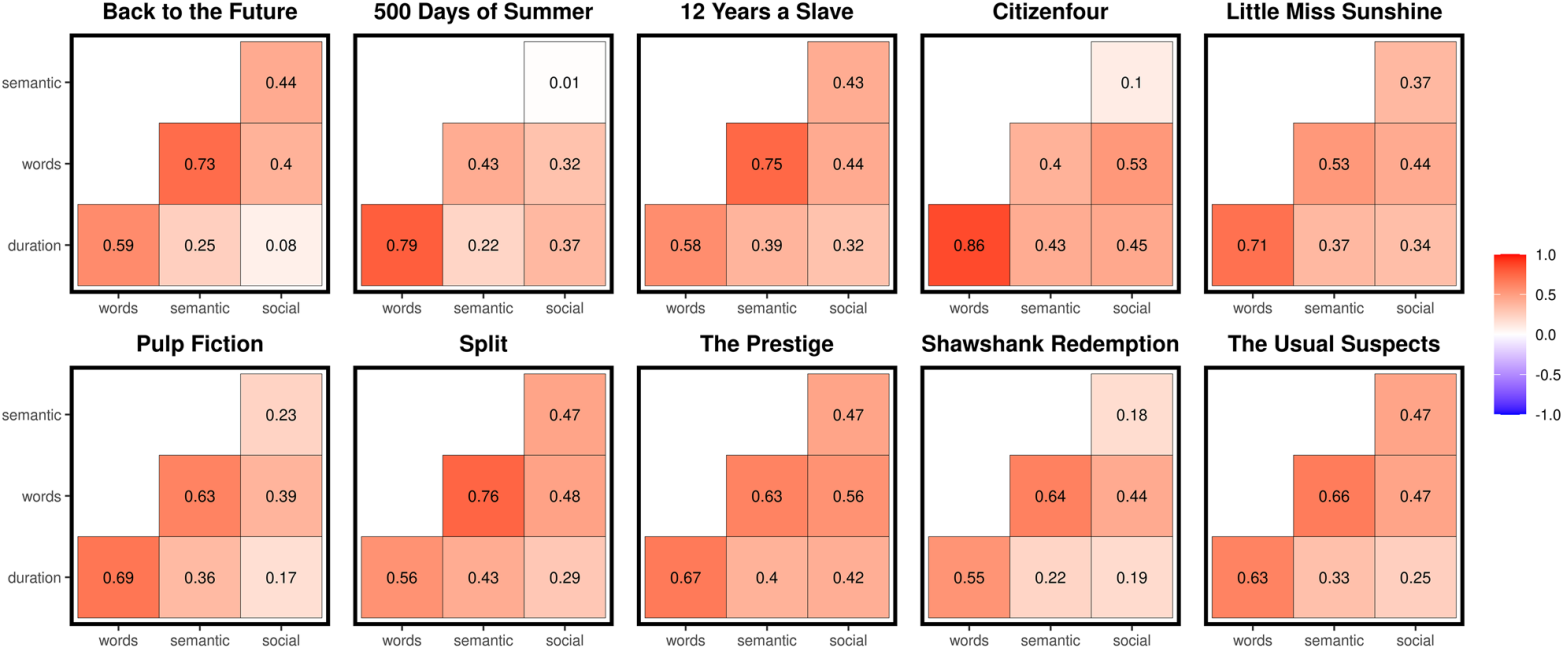
Event Property Correlations. Bivariate correlations between the number of words in an event, event duration, semantic rating, and social rating.

Correlations between the event properties, including semantic and social ratings, are shown in Fig. 5. Predictably, event duration and the number of words within an event were moderately to highly correlated (*r* = 0.55–0.86). Semantic ratings were positively correlated with number of words (*r* = 0.40–0.76), as were social ratings to a lesser extent (*r* = 0.32–0.56). This is unsurprising given that highly semantic events were defined as having new or informative verbal content and, to some extent, social moments in movies often rely on, or are supplemented by, verbal input. Although positively correlated, it was not the case that event ratings were simply proxies for duration or word quantity. Further, the semantic and social ratings did not capture the same event properties, as evidenced by the low to moderate correlations between the ratings (*r* = 0.01–0.47).

**Figure 6:**
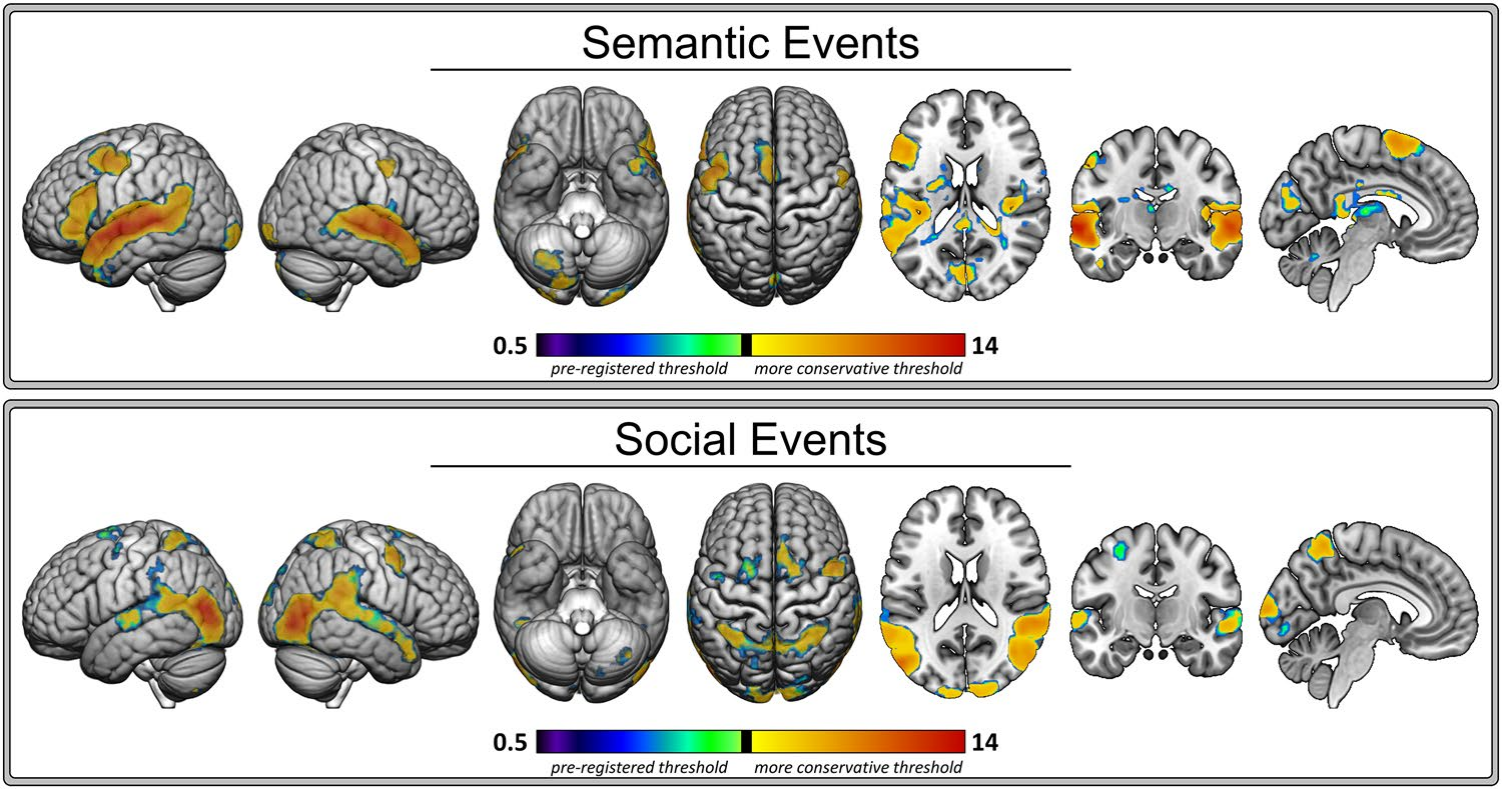
Events Analyses Results. Thresholded Z-score statistical maps showing the Semantic Events (top panel) and Social Events (bottom panel) results. All clusters survived the pre-registered cluster threshold. The clusters the survived an additional, more conservative threshold are indicated in yellow (lower) to red (higher). The clusters that did not survive the more conservative threshold are shown in purple (lower) to green (higher).

The events analysis results are shown in Fig. 6 and the coordinate information is provided in Table 4. The following sections provide an overview of the results for the pre-registered events analyses.

**Table 4:** Events results coordinate table.

Variable	Cluster size	Hem	Brain region peak voxel	Brain region highest overlap [%]	MNI coordinates		
					X	Y	Z
Semantic events	3690	L	Middle Temporal Gyrus	Middle Temporal Gyrus [25%]	− 56	− 33	− 0
	1916	R	Superior Temporal Gyrus	Superior Temporal Gyrus [34%]	65	− 1	− 2
	807	R	Precuneus	Putamen [8%]	23	− 46	16
	745	R	Cerebellum (Crus 2)	Cerebellum (VIII) [26%]	20	− 86	− 50
	354	L	Calcarine Gyrus	Cuneus [35%]	− 2	− 83	14
	232	L	Supplementary Motor Area	Supplementary Motor Area [87%]	− 5	1	74
	172	L	Lingual Gyrus	Inferior Occipital Gyrus [31%]	− 26	− 100	− 18
Social events	2545	R	Inferior Occipital Gyrus	Superior Temporal Gyrus [25%]	50	− 79	− 6
	1576	L	Middle Occipital Gyrus	Middle Temporal Gyrus [31%]	− 53	− 79	7
	1035	R	Superior Parietal Lobule	Superior Parietal Lobule [21%]	35	− 57	64
	601	L	Superior Occipital Gyrus	Calcarine Gyrus [19%]	− 5	− 101	6
	243	R	Middle Frontal Gyrus	Precentral Gyrus [65%]	50	2	54
	218	L	Cerebellum (VIIb)	Cerebellum (VII) [30%]	− 14	− 76	− 60
	178	L	Superior Frontal Gyrus	Precentral Gyrus [53%]	− 20	− 2	77
	159	R	Supplementary Motor Area	Supplementary Motor Area [59%]	8	10	77

This table was generated based on the pre-registered cluster threshold. Cluster size is determined by the number of 2 mm^3^ voxels. MNI coordinates correspond to the voxel with peak activation within each cluster. Voxels were defined as neighbours based on faces touching (NN = 1). Atlas labels are based on the Eickhoff-Zilles macro labels from the N27 (MNI space) atlas. Hem, Hemisphere; L, Left; R, Right.

##### Semantic events

**Figure 7:**
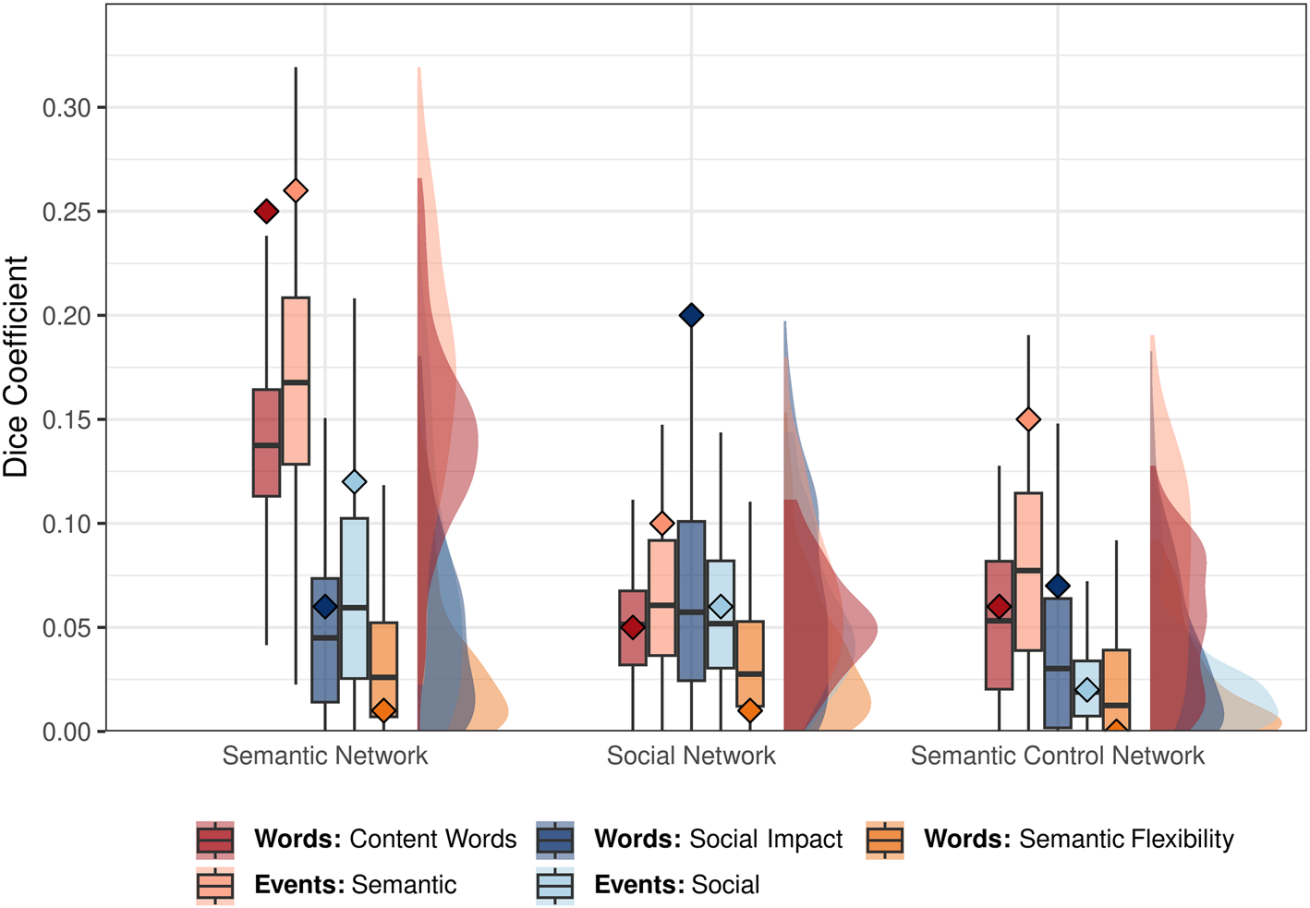
Network Overlap. The distributions of the subject-level overlap with the ALE-derived networks, measured with Dice similarity coefficient, are shown for each result: content words (dark red), semantic events (light red), social words (dark blue), social events (light blue), and semantically flexible words (orange). Diamonds indicate the Dice similarity coefficient between each network and the group-level results presented in Fig. 4 (words analyses) and Fig. 6 (events analyses).

It was expected that clusters of voxels showing increased activation in response to semantic events (plot-progressing, informative verbal or written scenes) would fall within the semantic brain network (Hypothesis 1.2a). In line with this prediction and the number of content words results, the Dice similarity coefficient was higher for the semantic events result and the semantic network (0.26; see light red diamonds in Fig. 7) than the social events results (0.12; see light blue diamonds in Fig. 7). Similar to the content words results, there was increased activation in large bilateral clusters centred around auditory cortices and extending from posterior to anterior superior temporal gyrus as the semantic content in events increased. The left hemisphere cluster included the middle portion of the temporal pole (i.e., lateral ATL) and extended posteriorly into inferior parietal lobule. There was also a large left inferior frontal gyrus cluster and a cluster in left supplemental motor area. Additional clusters of activation were observed in left cuneus and calcarine gyrus and inferior occipital gyrus and the right posterior lobe of the cerebellum. Subcortical activation positively co-varied with semantic event content in left putamen, thalamus, caudate nucleus, and a portion of the hippocampus.

##### Social events

It was expected that clusters of voxels showing increased activation in response to social events (scenes depicting on or off-screen interactions between/among characters) would fall within the social brain network (Hypothesis 1.2b). In line with this prediction, as the social content in events increased, there was increased activation in bilateral inferior parietal lobule (i.e., TPJ), right ATL extending posteriorly along superior temporal gyrus, left fusiform, precentral and middle frontal gyri, and supplementary motor area. Smaller clusters of activation in left calcarine and superior occipital gyri and a small cluster in the posterior lobe of the left cerebellum also positively co-varied with social event content. There were also prominent effects in bilateral lateral occipitotemporal cortex, which is typically associated with motion processing (V5/MT) and object recognition (LOC) rather than social cognition. Unlike the Social Impact words analysis results, social event content did not engage the left anterior temporal lobe. Further, the Dice similarity coefficient between this result and the social network was lower (0.06) than the Social Impact words result (0.20) and the semantic events result (0.10).

##### Scrambled events

Scrambled semantic and social ratings were used as a negative control condition for comparison with the critical predictors. There were no surviving clusters of activation positively associated with the scrambled ratings at either cluster correction threshold.

#### Correspondence with ALE-derived networks

Additional examination of the group-level maps was undertaken to further characterize the correspondence between the results and the ALE-derived networks (Fig. 1). The similarity between the ALE-derived networks and the group-level (RQ1) and subject-level (RQ2) results was quantified using Dice similarity coefficient (Fig. 7). With the exception of Semantic Flexibility, the semantic and social predictors engaged the semantic and social networks, respectively, as anticipated, although the social events result had substantially less overlap with the social cognition network than the social words result did. The subject-level Dice similarity coefficients are presented alongside the group-level values.

**Figure 8:**
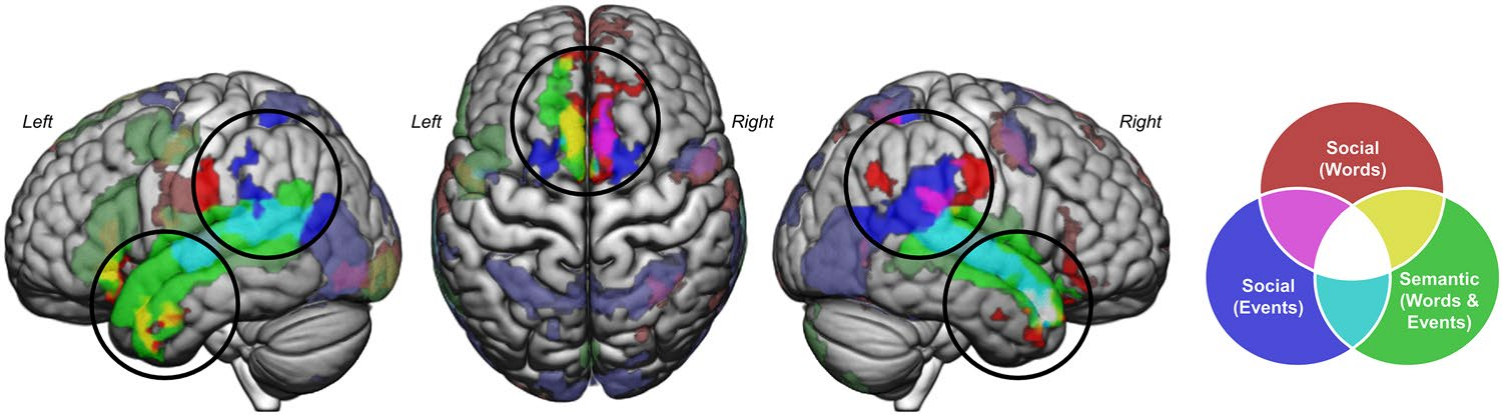
Conjunction of Group-Level Results. The conjunction of the semantic words and events (green), social words (red), and social events (blue) results. Overlap across all results is indicated with white shading. The black circles denote the following core semantic and social network regions, defined in Fig. 1: bilateral ATL, bilateral IPL, and SMA. Overlap outside these core areas has been desaturated.

A conjunction map showing the overlap of the group-level results within the core semantic and social network regions is presented in Fig. 8. There was overlap between the semantic and social words results and between the social words and social events results within the left and right portions of the supplementary motor area, respectively. Within IPL, both word and event-level social content engaged the same portion of right angular gyrus. Within the ATLs, the semantic and social words results overlapped in an anterior MTG portion of left ATL, and there was overlap across all results in a dorsolateral portion of right ATL. This pattern was further investigated within and across participants for Research Question 2.

### Research Question 2

#### Within-subject cognitive system overlap

**Figure 9:**
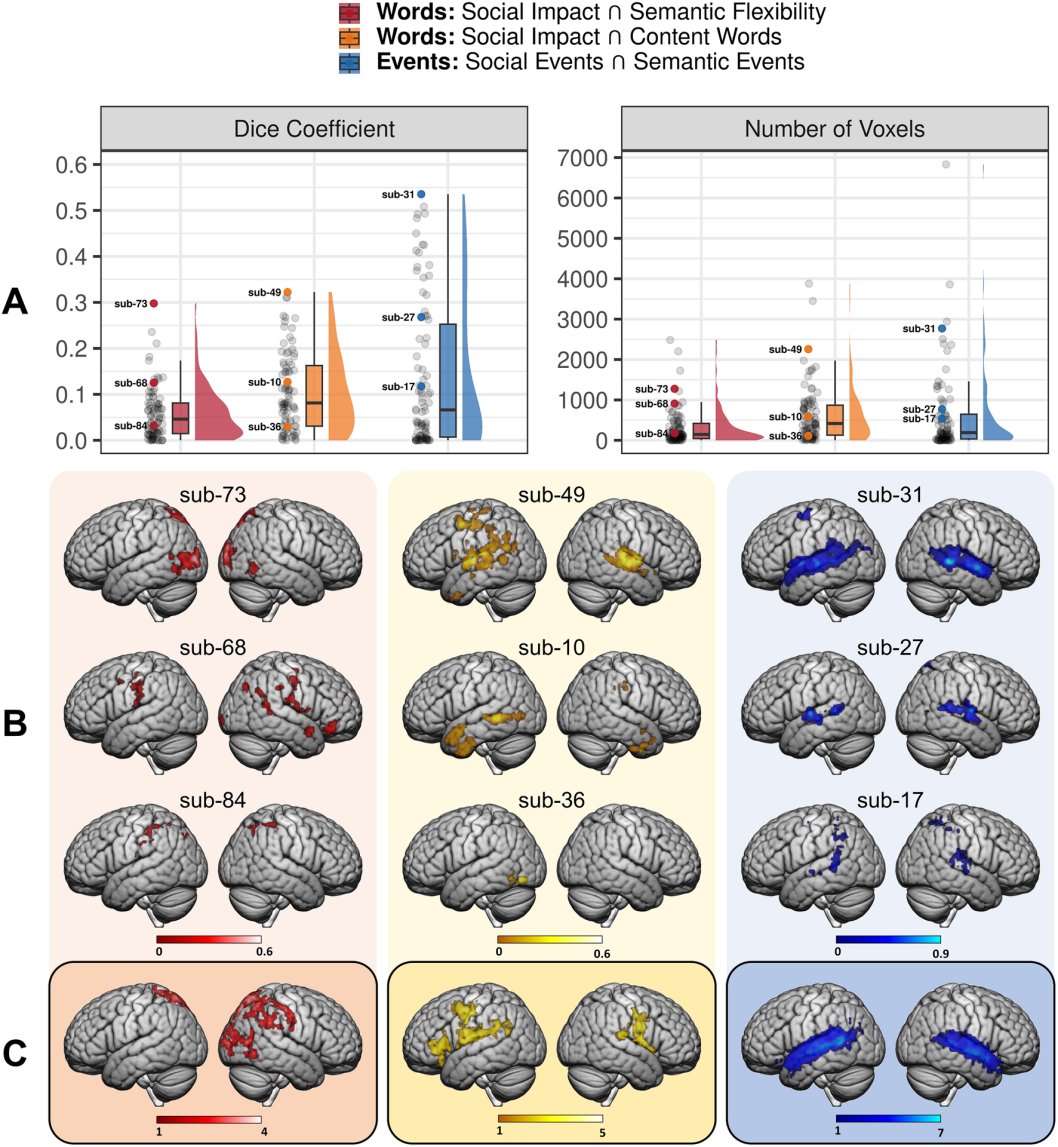
Subject overlap analysis results. (**A**): The within-subject overlap, measured with Dice Coefficient and Number of voxels, is shown for the Social Impact ∩ Semantic Flexibility results (red), Social Impact ∩ Content Words results (orange) and Social Events ∩ Semantic Events results (blue). Example subjects with high, moderate, and minimal overlap are highlighted for each overlap condition with subject labels to the left of points coloured according to the overlap condition (**B**): The normalized overlap maps for the example subjects highlighted in (**A**). (**C**): The cross-subject cognitive system overlap. The figures correspond to the Social Impact ∩ Semantic Flexibility (red), Social Impact ∩ Content Words (orange) and Social Events ∩ Semantic Events (blue) cross-subject overlap.

The subject-level statistical maps for each analysis were thresholded using a comparable approach for thresholding the group-level analysis with a cluster-forming threshold of *p* < 0.01 and an FWE-corrected threshold of *p* < 0.05. Cluster forming thresholds were determined by computing blur estimates for each subject from the residuals generated during the first-level analysis and used as inputs for 3dClustSim in AFNI resulting in subject-specific cluster tables. These thresholded subject-level maps were compared with the ALE-derived networks (Fig. 7) and with each other (Fig. 9).

There was considerable variability in the within-subject cognitive system overlap. This was evident by the range of Dice similarity coefficient values when comparing the subject-level results to the ALE-derived networks (Fig. 7) and when comparing the overlap between results within subjects (Fig. 9). There was modest overlap for Social Impact ∩ Semantic Flexibility: median Dice coefficient = 0.05 (range: 0–0.30), median overlapping voxels = 146 (range: 0–2481). Somewhat more overlap was observed for Social Impact ∩ Content Words: median Dice coefficient = 0.08 (range: 0–0.32), median overlapping voxels = 417 (range: 0–3879). Overlap for Social Events ∩ Semantic Events was particularly variable: median Dice coefficient = 0.07 (range: 0–0.54), median overlapping voxels = 191 (range: 0–6829). In each case, the distributions were strongly skewed such that many participants showed very little overlap and a small subset of participants showed moderate cognitive system overlap.

#### Cross-subject cognitive system overlap

In order to examine whether stable areas of overlap existed across participants, the subject-level overlap maps were used as inputs to a second-level random effects analysis. The pre-registered analysis plan was insufficiently clear on how this analysis would be carried out, resulting in ambiguity in how overlap would be quantified. As a result, we calculated overlap in two ways: (1) using binary overlap masks (e.g., areas within the thresholded subject-level statistical maps that overlapped between conditions) and (2) using statistical overlap masks (described in greater detail below). The former approach is not suitable for the pre-registered analysis strategy because a logistic modelling framework would better predict a binary (overlap or no-overlap) dependent variable. Instead of identifying consistent areas of overlap, an analysis using binary overlap masks produces an aggregate map of *any* overlap observed at the subject level, which is not the stated aim of Research Question 2. To avoid this issue, we opted to use method 2 (statistical overlap masks) for the analyses reported here. These masks were generated by first normalizing the thresholded subject-level statistical maps for each result by dividing by the max *t*-value, resulting in a range of values between 0 and 1. The normalized maps for each condition were then multiplied together (e.g., semantic events × social events), resulting in an overlap map where larger values indicated a greater response in *both* conditions. Finally, these normalized maps were used as inputs to the second-level random effects analysis with random effects of subject and movie as described in the pre-registration. Importantly, this analysis identifies whether there are voxels with values that are significantly greater than 0 associated with a given overlap type (e.g., overlap between semantic and social words). This is an anti-conservative test given the lack of negative values and the relatively large number of voxels where no overlap was observed (i.e., the value was 0) in the normalized overlap maps.

The cross-subject overlap coordinate information is characterized in Table 5 and overlap maps showing the most consistent loci of overlap between the cognitive systems are presented in Fig. 9C. The location of overlap varied considerably across participants, and in some cases (e.g., Fig. 9B**:** Social Impact ∩ Semantic Flexibility) isolated entirely different regions within participants. The most consistent location of overlap between the Semantic Flexibility and Social Impact words results was in right parietal lobule, right lateral occipitotemporal cortex, and portions of the occipital cortex. Overlap between the number of content words and Social Impact result was most consistently localized to left inferior frontal gyrus and bilaterally anterior to the Sylvian fissure through the pre and postcentral gyri and rolandic operculum into posterior superior temporal gyrus and supramarginal gyrus. As illustrated in Fig. 9B, there was within-subject overlap in left ATL in some participants (sub-49, sub-10), but this was not a consistently identified location of overlap across participants. Overlap between the social and semantic events results consistently occurred along bilateral superior temporal gyri, extending from the superior portion of ATL posteriorly into supramarginal gyri. This may be driven by the amount of verbal input (i.e., number of words) which was correlated with the event ratings.

**Table 5:** Cross-subject overlap coordinate table.

Variable	Cluster size	Hem	Brain region peak voxel	Brain region highest overlap [%]	MNI coordinates		
					X	Y	Z
Social Impact ∩ Semantic Flexibility	2105	R	Superior Parietal Lobule	Superior Parietal Lobule [15%]	20	− 73	66
Social Impact ∩ Content Words	1181	L	Superior Temporal Gyrus	Postcentral Gyrus [22%]	− 50	− 40	22
	831	R	Rolandic Operculum	Rolandic Operculum [27%]	59	− 12	14
Social Events ∩ Semantic Events	2032	L	Middle Temporal Gyrus	Middle Temporal Gyrus [38%]	− 62	− 51	5
	1661	R	Middle Temporal Gyrus	Superior Temporal Gyrus [48%]	50	− 33	− 0

Cluster size is determined by the number of 2 mm^3^ voxels. MNI coordinates correspond to the voxel with highest statistical value within each cluster. Voxels were defined as neighbours based on faces touching (NN = 1). Atlas labels are based on the Eickhoff-Zilles macro labels from the N27 (MNI space) atlas. Hem, Hemisphere; L, Left; R, Right.

## Discussion

### Overview

The present study investigated the neural basis of semantic and social processing during movie-viewing, which provides a rich estimation of the multimodal environment in which language use and social interactions take place^79^. Current neurobiological models of the semantic and social cognition systems were derived from experimentally controlled stimuli presented in random order without larger-scale context. Naturalistic stimuli are less constrained, tending to evoke highly stable patterns of brain activation in regions different to those identified in minimalist experiments, allowing for different insights into fundamental aspects of human cognition^80^. In order to comprehensively capture semantic and social content within the movies, word and event-level predictors were generated and used for analyses. The goals of the study were twofold. The first aim was to test the degree to which semantic and social content was processed within each network. The second, complementary aim was to test the degree to which the semantic and social systems evidence shared processing in the same regions or domain-general hub, given the conceptual and neural overlap between these systems.

In line with our predictions (hypotheses 1.1a and 1.2a), word and event-level semantic content isolated a highly convergent, largely fronto-temporo-parietal network, despite measuring semantic content in different ways. Frequent, semantically diverse language (estimated by positive Semantic Flexibility scores) did not co-vary with activation in semantic control regions, counter to our expectations (hypothesis 1.1c). An increase in both word and event-level social content engaged portions of the social network, but the word-level social content engaged a network of regions more similar to the social network highlighted in Fig. 1 than the event-level social content did. That is, the evidence is consistent with our social word predictions (hypothesis 1.1b) and partially consistent with our social event predictions (hypothesis 1.2b). There were no positive associations with the scrambled ratings, increasing confidence that we report meaningful associations with the semantic and social event ratings. There were stable, cross-subject loci of overlap between semantic and social processing, although these loci differed between the words and events analyses, providing partial evidence of shared processing (hypothesis 2.1a). Specifically, the cross-subject word-level semantic and social overlap was localized to regions which predominately fell outside the semantic network, providing no support for the hypothesis that semantic regions would be engaged. The event-level semantic and social overlap, however, engaged much of the semantic network, providing support for the hypothesis. There was no evidence of consistent overlap between the Semantic Flexibility and Social Impact words results within the semantic control network, counter to our predictions (hypothesis 2.1b). The subject-level results were highly variable, which precluded a formal test of the non-overlap predictions at the subject-level (hypotheses 2.2a and 2.2b), but a conjunction of the group-level results indicated that there were both overlapping as well as non-overlapping but proximal areas within core semantic and social brain network regions that are engaged by semantic and social word and event-level content embedded in movies. This was most prominent in bilateral ATL: predominately verbal semantic and social content engaged a shared portion of left ATL in anterior MTG, whereas the superior portion of right ATL was engaged by word and event-level semantic and social content. In addition, there was evidence of graded functioning within ATL such that social words minimally engaged the superior, or dorsolateral, portion of ATL which was robustly engaged by semantic content. All analyses and interpretations given to results were pre-registered prior to conducting the study. The following sections provide an overview of each content type, indicating, where relevant, when an interpretation that was not pre-registered is provided.

### Semantic content

Activation in semantic and semantic control network regions positively co-varied with an increase in both word and event-level semantic content in complex movie stimuli. This network included the core regions highlighted in the meta-analytic map shown in Fig. 1: bilateral ATL, bilateral middle temporal gyrus, left supplementary motor area, left IPL, semantic control regions in left inferior frontal gyrus and posterior middle temporal gyrus, and minimally included activation outside these networks. We made no specific predictions about the recruitment of the semantic control system in response to content words or highly semantic events because we had no corresponding measure of how cognitively demanding the words or events were (with the exception of Semantic Flexibility, discussed below). However, engagement of the semantic control system is routinely found in studies of semantic cognition and—unless they are explicitly removed—these control regions are found in meta-analyses of semantic cognition^39,81^. For the present study, semantic control regions were subtracted from the semantic system in order to distinguish automatic semantic processing regions from those regions which are involved in more effortful or controlled semantic processing. This distinction may be useful for some experimental contexts, but the present results suggest that semantic control processes are an integral part of how the semantic system operates in naturalistic comprehension. The Semantic Flexibility results (discussed below) further suggest that semantic control engagement in naturalistic contexts may be quite different from the manipulations used in minimalist experiments. That is, in naturalistic contexts, semantic control may be particularly important for comprehending semantically rich moments in the narrative (i.e., a large number of content words or highly semantic events) rather than resolving the kinds of lexical or syntactic ambiguities that are studied in minimalist experiments (because those are resolved by the context).

The fronto-temporo-parietal network observed here appears to have considerable overlap with the proposed ‘universal language network’. This network has been identified across diverse languages and is thought to include language selective brain regions^82^, although some argue that this network is the by-product of averaging over varied language representations, thus obscuring the neurobiology of the distributed language system^83^. Apparent convergence with the topography of this network is notable because the ‘universal language network’ was derived with a different naturalistic paradigm: story excerpts compared with acoustically degraded audio (or unfamiliar language) to identify the network of regions sensitive to language. The present study used continuous measures of high versus low semantic content to isolate regions that are particularly sensitive to an increase in semantic content. This focus on semantic content may explain why we observed more activation in the lateral portion of the ventrolateral ATL, which was only present in some of the language networks (including English) reported by Malik-Moraleda et al.

Correspondence with the ‘universal language network’ suggests that there is a robust language comprehension network that is engaged across naturalistic contexts, including narratives and movies. Indeed, this network is well positioned to serve this purpose with structural connections between orbitofrontal cortex and temporal pole (uncinate fasciculus), inferior frontal cortex to posterior superior temporal cortex (arcuate fasciculus) and inferior parietal lobule (extreme capsule)^84^. Although not part of the semantic or language network, activation in right posterior cerebellar lobe may be similarly driven by the structural connections projecting into perisylvian language areas, suggesting a possible role in general language processing^85^. A similar network topography has been reported in other studies that used naturalistic stimuli, predominately narratives and natural language^86,87^. Individual studies using minimalist stimuli tend to report highly focal results, however, aggregation of study-level data via meta-analysis reveals a network with similar topography to that observed here^39,81^, although naturalistic stimuli tend to evoke a more bilateral network.

The semantic content in complex movie stimuli was quantified using a word-level and an event-level definition, and, although both measures were used as proxies of semantic processing, it is nonetheless striking that they isolated a highly convergent fronto-temporo-parietal network given the conceptual differences between the measures. The word-level measure (content word quantity) was agnostic to the context of the event or larger narrative, but that information directly informed the event-level measure (subjective ratings). The latter was sensitive to both the local context, in that ratings were given to events segmented based on the progression of the narrative, and the global context, in that ratings were given consecutively allowing for prior information to impact perception of the event. It is thus notable that the word and event semantic content predictors engage a highly similar network given the impact of context on conceptual representations^88^. Conceptually and practically, both measures were strongly impacted by the total number of words, which was evidenced by the moderate-to-high correlations between number of words and semantic event ratings across movies. In naturalistic communication, especially in scripted narratives, semantic content is inherently related to verbal input (though they are not identical: semantic ratings also captured narrative moments with non-spoken information or moments in which highly important information is conveyed using few words). Statistically removing that association would create minimally interpretable event ratings; a different approach is needed for studying naturalistic semantic processing independent of verbal processing. For the present study, a more useful approach is to consider the word-level and event-level results together, noting their similarities and differences.

There were minor differences in the topography of the word-level and event-level semantic content networks. The semantic event network engaged a larger cluster in left inferior frontal gyrus and supplementary motor area, as well precuneus, middle cingulate cortex and surrounding subcortical structures (putamen, thalamus, caudate nucleus), and left inferior and middle occipital gyrus. This may reflect differences in word versus event processing. For instance, the occipital activation observed for highly semantic events is consistent with the fact that linguistic content could be presented via spoken or written language. In addition, differences in temporal receptive windows drive regional recruitment in response to word, sentence, or paragraph presentation^52^. The latter two contexts engage a large left inferior frontal region as well as posterior cingulate and precuneus, which we similarly observe in the network that co-varies with highly semantic event content. Processing movie events, which incorporate context in much the same way as sentences or paragraphs, could then drive engagement of regions which allow preceding context to have a greater influence on the integration of information. In addition, or alternatively, event content ratings may have been influenced by other types of information (e.g., emotional information), so the resulting network of regions may reflect sensitivity to both semantic and correlated information.

In a recent study using similar word-level predictors but with an audiobook stimulus, engagement of the same lateral portion of ATL was not observed in response to an increase in the quantity of content words^89^. Instead, the temporal pole, including ventrolateral ATL, was active when there was a decrease in content words. This was thought to be driven by the fact that the speech rate, and therefore approximate quantity of words, tends to be fairly consistent throughout an audiobook. The ventrolateral hub then is still engaged in semantic processing and may use the relative decrease in new, plot-progressing information to integrate the current information with the prior knowledge of the narrative. This is not the case for movies, which can have periods that are primarily (or entirely) visual, when minimal verbal information is presented. Both studies used a parametric modulation approach, but in the audiobook context, the ‘low semantic’ condition still contained words (predominately closed class words) whereas the same condition in the present study likely contained no words at all (scenes with only visual information). In measuring regional covariation in response to high relative to low semantic content, it appears that ATL activation does not selectively co-vary with an increase in content words in the audiobook case where the speech rate is consistent even for the low semantic periods. Conversely, in the movie case where ‘low semantic’ means limited to no verbal input, we see ATL activation co-vary with an increase in semantic content. This may indicate important differences in how unimodal (e.g., audiobook) and multimodal (e.g., movie) narratives engage the same cognitive system, which needs to be considered when defining research questions or adapting paradigms. Anticipating this difference between audiobooks and movies, for instance, the present study used summed factor scores instead of mean factor scores to better capture the total amount of each content type. Using means instead of sums appears to better approximate the relative amount of these properties when the speech rate is consistent, as in the audiobook case.

We speculate that ventrolateral ATL, in particular the lateral portion of this hub, is engaged by internal, or endogenous, semantic processing required for updating and processing the ongoing narrative, in contrast to the exogenous process of comprehending the stimulus. With a longer temporal receptive window for accumulating and integrating information^51,52^ and with functional connections to the broader default mode network (DMN)^90,91^, this region is well-suited to serving this role. The DMN, which is active in the presence and absence of external input, facilitates the construction of an continuous coherent internal narrative by relying on episodic and semantic memory^92^. This is observed across naturalistic comprehension contexts. Intact story comprehension elicits robust, cross-subject stimulus induced changes in connectivity between the posterior cingulate cortex, a core DMN region, and anterior MTG (overlapping with the ventral portion of the hub)^93^, and activation in the DMN covaries with high-level perception of narrative features during movie-viewing^94,95^. We suggest that functional connections between the default mode network and ventrolateral ATL drive narrative integration and support endogenous semantic processing of the narrative content. The endogenous processing demands placed on this hub are poorly approximated by the relative amount of semantic input. Instead, the dorsolateral ATL appears to be particularly sensitive to the quantity and informativeness of the input as evidenced by greater activation in this region in both word and event results of the present study and in the ‘universal language network'.

### Semantic Flexibility

Counter to our pre-registered hypothesis, there was no evidence of increased engagement of semantic control network regions as semantically flexible word-level content increased. This suggests that processing words with positive Semantic Flexibility scores, which are associated with more frequent, less concrete, and more semantically diverse language, does not require more semantic control. We expected words with high semantic diversity to place additional demands on the control system due to the need to select from one of several possible meanings that best fit the context^63^. This prediction was based on prior studies, which tended to use highly decontextualized stimuli in which meaning selection did not benefit from the context provided in a narrative. During naturalistic language comprehension, highly semantically diverse language appears to be disambiguated by the preceding context without requiring engagement of the semantic control system. These results suggest important deviations from single-word and sentence level investigations of semantic diversity and ambiguity resolution more generally. Similar to the present study, an increase in bilateral parietal and occipital activation was observed in a previous study as positive Semantic Flexibility scores increased^89^.

### Social content

Recent evidence suggests that social knowledge is subsumed within the semantic system and, like other types of semantic information, is processed in the transmodal ventrolateral ATL hub^7,30^. In this view, all kinds of social stimuli are processed within the semantic system, ranging from social concepts, which have consistently been shown to recruit portions of left ATL^14,20,26^, to more abstract social processes such as mentalizing, which have not been as thoroughly investigated (but see^30^). The present study enabled a direct test of this claim using naturalistic movie stimuli, which better approximate real-world socio-cognitive processing, across two social contexts: (1) social words, estimated using Social Impact factor scores, and (2) social events, using subjective event-level ratings. We hypothesized that the social network shown in Fig. 1 would be engaged by both content types, in particular within the core regions highlighted in the figure. The results provide partial support for this claim.

An increase in highly social and emotionally arousing words engaged much of the social cognition network: dorsomedial prefrontal cortex, bilateral IFG, superior frontal gyrus, supramarginal gyrus, precuneus, bilateral ATL, and left IPL, with minimal engagement of bilateral MTG. These results broadly align with the pre-registered predictions, and are similar to the regions identified in separate meta-analyses of social compared to non-social concepts^96,97^. Unexpectedly, however, activation in left IPL did not co-vary with an increase in Social Impact. In addition to the core social cognition regions, an increase in word-level social content co-varied with activation in precentral and postcentral gyri, middle cingulate cortex, and left inferior occipital gyrus. Activation in ventrolateral ATL co-varied with an increase in social and emotional language. This provides critical support for the claim that social processing is supported by the semantic system^7^, but it is important to consider the nature of the contrast. The predictor in this analysis was the socialness of the words, with the quantity of semantic content statistically controlled by using residual scores, so the analysis should not identify regions that are responsive to general semantic or language comprehension. That is, if activation of the ventrolateral ATL hub is primarily driven by the amount of semantic content, then it should *not* co-vary with Social Impact after controlling for semantic content.

If ventrolateral ATL activation during periods of high Social Impact cannot be attributed to an increase in general semantic content, what is driving this effect? Engagement of this hub for processing social relative to non-social word-level content may suggest an increased sensitivity to social information, at least in naturalistic contexts. Alternatively, and building upon our claim about ventrolateral ATL, word-level social processing may drive greater engagement of ventrolateral ATL due to a greater need for endogenous semantic processing. This may be a consequence of the general role of the DMN in social processing^98^ or may be due to the nature of movies in which social information is particularly salient and important to the narrative.

Although the word-level analysis used orthogonalized factor scores, a ‘pure’ social factor did not emerge from the PCA. Instead the Social Impact factor was driven by socialness and emotional arousal, making it hard to disentangle the social versus emotional effects. In complex, naturalistic stimuli, however, social and emotional content are likely to be at least moderately correlated. Highly social moments are often emotional, and, inversely, emotional moments frequently play out in social interactions between characters, given the likely oversampling of social content in compelling storytelling. Further, conceptual representations are not static^88^, but the word-level predictor treated the words as independently sampled from the narrative, a limitation that the event-level predictor directly addressed.

In accounting for the impact of context on conceptual representations, the event-level analysis may have better captured the kind of socio-cognitive processing typically isolated in studies of social cognition. Although social event ratings were moderately correlated with word quantity, the social event predictor also captured non-linguistic content, as intended. Highly interpersonal moments in a movie may contain few, if any, words and are separate from the linguistic content present in the event. Conceptually, the word and event predictors could capture different properties of the underlying stimulus, although prior work looking at the correspondence between word-level and passage level emotion ratings suggests otherwise^99^.

Highly social events engaged a network that only partially overlapped with the word-level social content network and included different core social cognition regions. Activation along bilateral superior temporal gyrus extending posteriorly into bilateral lateral occipitotemporal cortex and left angular gyrus co-varied with the social content in events. Processing dynamic social events appears to engage motion processing areas in middle temporal visual motion area (MT), face and object recognition areas in the lateral occipital cortex, and superior temporal sulcus, which may aid in face and body perception^100–103^. The ATL hub was not recruited during social event processing, providing counterevidence against the claim that general social processing recruits the domain-general semantic hub^30^. However, it may be that the ventrolateral hub was equally engaged by highly and weakly social events, and, unlike the word-level results, did not evidence increased sensitivity to social events.

The overlap between the social and semantic control system has been interpreted to suggest that socio-cognitive processing places increased demands on the semantic control system^20,31^. Support for this claim in the present study is mixed. Words that were more social and emotionally arousing (i.e., higher Social Impact) engaged the semantic control network in bilateral inferior frontal gyri, but this was not observed for highly social events. An important consideration in weighing the evidence is the degree to which the word and event-level predictors may have had different control demands that are hard to quantify in naturalistic stimuli. Alternatively, social event-level information may be more readily understood than word-level information. This inference is not without precedent. Many social phenomena studied out of context have been shown to increase general control demands. Processing embedded mental states (e.g., Marty *understands* that Doc *believes* that reading the letter would change the future), for instance, is effortful in a sentence or passage context^104,105^, but is readily understood, and even enjoyable, in the narrative context^106^. Humans process information well when presented in narrative format^107^, which movies provide. Socio-cognitive processing may engage the semantic control system in experimental paradigms that present de-contextualised stimuli in a random order, but not in a rich narrative context or during naturalistic social processing. Taken together, we do not find strong support for the claim that socio-cognitive processing increases semantic control demands. Prior studies isolating specific social processes that found support for this claim are challenged by the ease with which humans engage in these processes in naturalistic contexts.

### Shared processing

One of our study aims was to investigate engagement of the semantic and semantic control networks in processing social knowledge. At the group-level, there was evidence of shared processing within the ATLs such that (1) activation in the same anterior MTG portion of left ATL was associated with verbal semantic and social content (i.e., social words) and (2) activation in the anterior STG portion of right ATL was associated with word and event-level semantic and social content. This pattern is consistent with prior research reporting an ATL asymmetry with a left hemisphere bias for verbal content and right hemisphere bias for non-verbal content^108,109^. We further examined the consistency of this overlap in preregistered subject-level analyses.

The distribution of subject-level overlap, quantified using number of voxels and Dice Similarity Coefficient (Fig. 9), suggested that cross-system overlap exists, but its location was highly variable across subjects and the median values tended to be modest. As shown in Fig. 7, even the overlap between the subject-level results and the ALE-derived networks was inconsistent: some participants had moderate overlap but many participants had minimal to no overlap. Further, the subject-level median Dice similarity coefficient values were almost always well below the group-level values, suggesting that idiosyncratic sources of noise were captured at the subject-level that were averaged out at the group-level. It is possible that the variability is a product of statistical thresholding such that sub-threshold voxels are removed resulting in the impression of no overlap. When the full subject-level statistical maps are used in a group-level analysis, these sub-threshold voxels are accounted for in the analysis rather than removed altogether, providing a better estimate of the networks engaged in processing each content type. In addition, subject-level analyses benefit from robust localizers that consistently identify the same brain network or region across individuals. The predictors used in the current study were not validated localizers known to elicit the targeted cognitive processes. Moreover, the predictors were embedded in a naturalistic paradigm, itself a significant source of noise. Given this, the subject-level variability is not surprising. The use of alternative subject-level analysis approaches, such as using well-validated functional localizers to define functional regions of interest that reliably respond to a given content type^110^, might provide better estimates of within-subject overlap between semantic and social processing. Although we did not find strong support for the preregistered hypothesis regarding shared processing within semantic and social brain regions at the subject-level, we suggest that this is driven, in part, by the predictors used and encourage future studies to make use of robust localizers to investigate this claim.

Despite the within-subject variability, there were reliable cross-subject loci of overlap that indicate shared processing between the semantic and social systems. Interestingly, however, the consistent areas of overlap differed for the word and event predictors. As discussed in the sections above, the semantic word and event predictors both captured a similar fronto-temporo-parietal network, but the social word and event predictors captured different networks. The subject-level overlap between the word and event predictors thus resulted in different loci of overlap with the word-level predictors localized to bilateral precentral gyri and left inferior frontal gyrus and the event-level predictors localized to bilateral superior temporal and supramarginal gyri. These results provide a useful complement to the group-level results because they highlight the voxels that consistently respond strongly to *both* content types within subjects. The results suggest that, at the subject-level, social and semantic content both engage regions within the semantic network but word-level and event-level social content engages different regions within the semantic network.

The consistent loci identified for the Semantic Flexibility and Social Impact words results were largely posterior regions in right inferior parietal lobule and occipitotemporal cortex. Importantly, contrary to our prediction, an increase in Semantic Flexibility did not engage the semantic control network, so the overlap between these variables is difficult to interpret because the predictor did not isolate the semantic control network within subjects. The group-level Social Impact results suggest at least partial engagement of the semantic control network in left IFG, but a stronger narrative-level manipulation of semantic control demands is required to investigate the subject-level overlap between the social and semantic control systems.

## Conclusions

Naturalistic neuroimaging data provide an exciting and rich basis for studying the neural basis of human cognition. However, this richness also makes them particularly vulnerable to adjusting analysis strategies and constructing post hoc explanations, which is common in whole-brain neuroimaging. The analyses and hypotheses described in the present study were based on well-defined theories of semantic and social cognition (and how they might interact) and pre-registered to maximize transparency about the analysis plan (and any deviations) and distinguish a priori hypotheses from post hoc speculations based on the results.

The results suggest that, during naturalistic movie viewing, increases in semantic content are associated with increased activation in the semantic and semantic control networks, displaying a fronto-temporo-parietal topography highly similar to the universal language network^82^. There is evidence of a hub architecture, consistent with the graded hub hypothesis^24^, but the ATL subregions appear to serve different functions during naturalistic comprehension. Left ATL was predominately engaged by verbal semantic and social content within the movies, whereas right dorsolateral ATL was engaged by both verbal and non-verbal semantic and social content. We suggest that the dorsolateral ATL is sensitive to the quantity and informativeness of the input, as evidenced by robust activation during language comprehension, whereas the lateral portion of ventrolateral ATL hub may be also important for endogenous semantic processing—updating and processing the ongoing narrative—leveraging this region’s functional connections with the default mode network. Word, but not event-level, social content engaged the ventrolateral ATL, perhaps because social content is particularly important for movie narratives and consistent with the role of this region in endogenous semantic processing. Social events engaged a network topographically more similar to the social cognition network, with activation in bilateral TPJ. Although portions of the semantic network (ATL, right IPL) were engaged by social content and these regions overlapped at the group level, the subject-level overlap analyses suggest limited cross-subject consistency. These results are a step toward integrating theories of word-level semantic cognition with theories of narrative comprehension and understanding the relationships between social and semantic cognition.

### Supplementary Information

Supplementary Figures.

## Data Availability

The raw and preprocessed data are publicly available on OpenNeuro (https://openneuro.org/datasets/ds002837/versions/2.0.0). All additional data and materials generated as part of this project are shared publicly on OSF: https://osf.io/dur8a/. This includes the final processed neuroimaging data and the movie annotations indicating the onset and duration of the events within each movie and the corresponding semantic and social content scores for each event.

## Code availability

All analysis code associated with this project is shared on OSF: https://osf.io/dur8a/.
