# Supplementary material for: The neural basis of naturalistic semantic and social cognition
Source: Sci Rep. 2024 Mar 21;14:6796. doi: 10.1038/s41598-024-56897-3 (PMC10957894; doi:10.1038/s41598-024-56897-3)
Supplement: Supplementary file 1 — Supplementary Figures. [file 41598_2024_56897_MOESM1_ESM.docx]

# Supplementary information

**
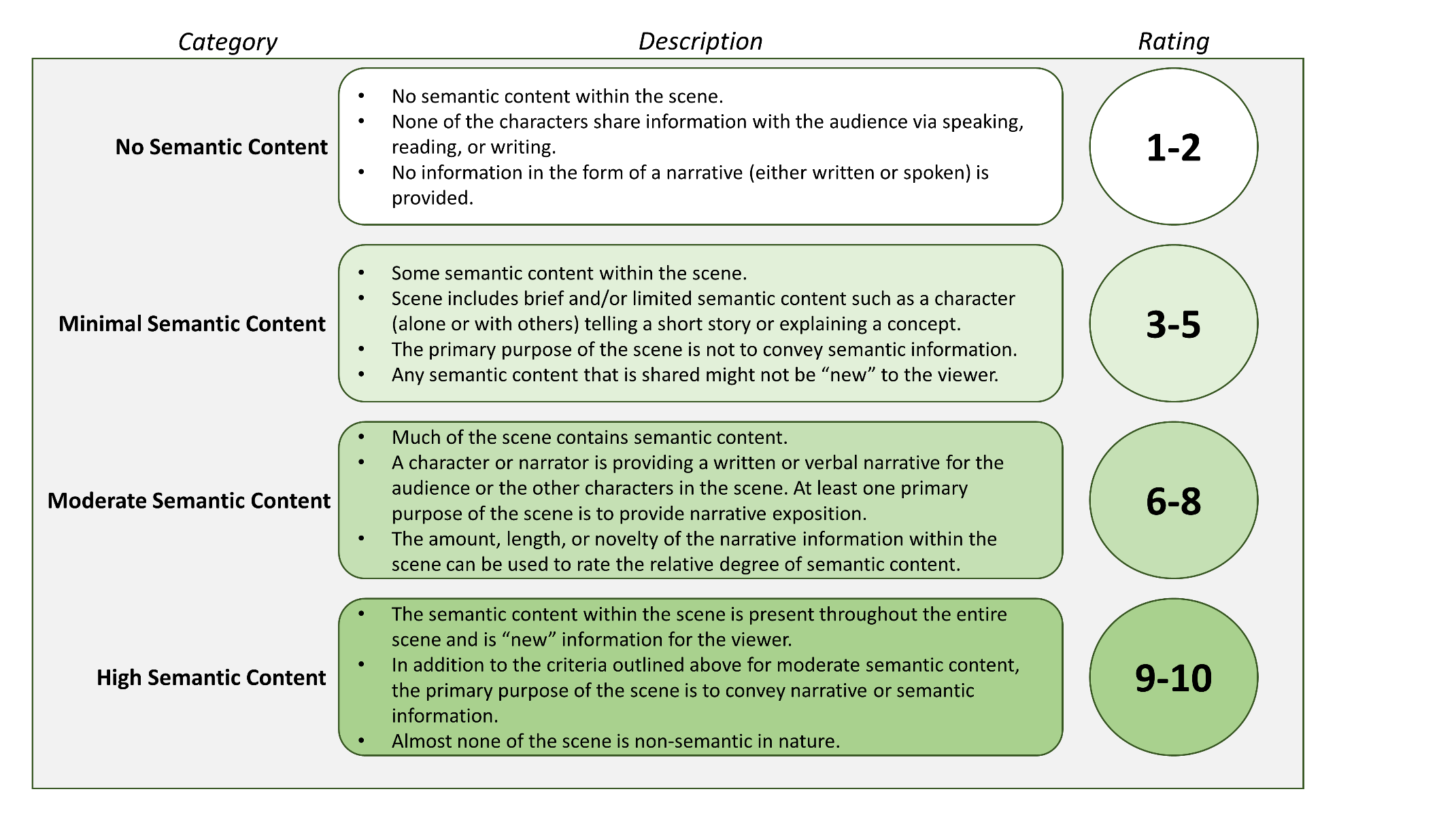
**

**Supplementary Figure 1. Rubric for how semantic content was rated for each minor event.** The rubric defines four broad categories (right) that are described (middle) and associated with a range of possible ratings to assign to a given event (left).


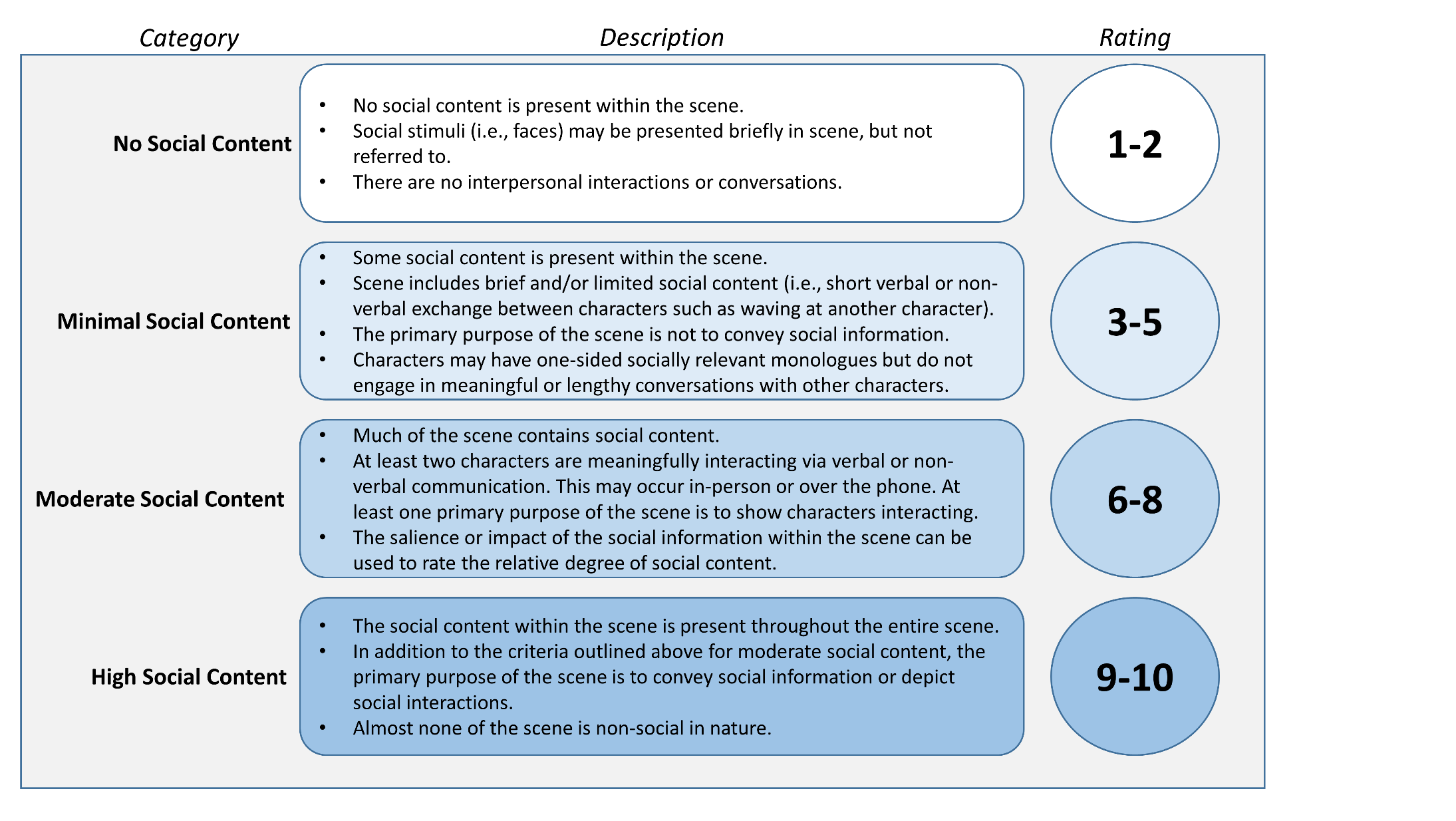


**Supplementary Figure 2. Rubric for how social content were rated for each minor event.** The rubric defines four broad categories (right) that are described (middle) and associated with a range of possible ratings to assign to a given event (left).
